# Discovery of tissue-specific exons using comprehensive human exon microarrays

**DOI:** 10.1186/gb-2007-8-4-r64

**Published:** 2007-04-24

**Authors:** Tyson A Clark, Anthony C Schweitzer, Tina X Chen, Michelle K Staples, Gang Lu, Hui Wang, Alan Williams, John E Blume

**Affiliations:** 1 Affymetrix, Inc., 3420 Central Expressway, Santa Clara, CA 95051, USA

## Abstract

**Background:**

Higher eukaryotes express a diverse population of messenger RNAs generated by alternative splicing. Large-scale methods for monitoring gene expression must adapt in order to accurately detect the transcript variation generated by this splicing.

**Results:**

We have designed a high-density oligonucleotide microarray with probesets for more than one million annotated and predicted exons in the human genome. Using these arrays and a simple algorithm that normalizes exon signal to signal from the gene as a whole, we have identified tissue-specific exons from a panel of 16 different normal adult tissues. RT-PCR validation confirms approximately 86% of the predicted tissue-enriched probesets. Pair-wise comparisons between the tissues suggest that as many as 73% of detected genes are differentially alternatively spliced. We also demonstrate how an inclusive exon microarray can be used to discover novel alternative splicing events. As examples, 17 new tissue-specific exons from 11 genes were validated by RT-PCR and sequencing.

**Conclusion:**

In conjunction with a conceptually simple algorithm, comprehensive exon microarrays can detect tissue-specific alternative splicing events. Our data suggest significant expression outside of known exons and well annotated genes and a high frequency of alternative splicing events. In addition, we identified and validated a number of novel exons with tissue-specific splicing patterns. The tissue map data will likely serve as a valuable source of information on the regulation of alternative splicing.

## Abstract 2

Comprehensive exon microarrays with a simple intra-gene normalization algorithm were used to detect human tissue-specific alternative splicing events, suggesting significant expression outside of known exons and well annotated genes and a high frequency of alternative splicing events.

## Background

Alternative splicing dramatically expands the protein coding potential of higher eukaryotes. Current estimates suggest that greater than 60% of human genes have more than one isoform. The expression of specific transcripts is regulated in developmentally and tissue-specific manners (reviewed in [1]). Alternatively spliced isoforms from the same gene can produce proteins with different properties and distinct functions. In one example, two mRNAs transcribed from the *bcl-x *gene that utilize different 5' splice sites result in proteins that have antagonistic functions. The short form of *bcl-x *promotes apoptosis, while the long form inhibits cell death [2]. Errors in mRNA processing have been associated with cancer and other human diseases [3]. It has been estimated that 15% of disease-causing point mutations in humans affect sequences that regulate splicing [4]. In the pharmaceutical industry, alternative splicing is often overlooked but could be important to drug discovery programs. New targets previously missed, or additional isoforms of existing drug targets could be discovered, thereby increasing the pool of targets for new drug screening. Valuable information can be obtained by studying the levels of individual transcripts. It is possible that previously unknown or unstudied transcript variants for genes could provide new insight into biological function or provide new targets for mechanism of action studies and drug development. Current large-scale methods for monitoring expression must evolve to take into consideration the richness of transcript variation.

Much of what is currently known about the mechanism and regulation of splicing has come from research of a relatively small number of splicing reporter constructs and a handful of regulated exons [1]. While this focused approach has been initially informative, it is not yet clear if it will generalize to other splicing events or the splicing apparatus as a whole. Because of differences in context and surrounding sequence each splicing event is essentially unique. With so many unique events, the ability to study the splicing of many events in parallel should provide valuable insight into the mechanism of splicing and the regulation of alternative splicing.

To date, most of the studies of genome-wide alternative splicing have relied on predictions of splicing events based on expressed sequence tag (EST) or mRNA sequences [5,6]. However, using ESTs to study splicing poses several challenges, including a bias towards the ends of transcripts, poor coverage of many tissue types, differences in library construction protocols, and poor representation of low abundance transcripts [7]. In addition, some EST libraries also contain poor quality sequence, genomic contamination, and mis-spliced RNAs, which can confound the results. While efforts have been made to estimate expression patterns and find tissue-specific splicing events using EST frequency and library source information [8-10], the examination of EST sequences is not ideal for studying alternative splicing. Furthermore, it is not practical to extend sequencing based approaches to cover all tissues, disease states, and stages of development.

DNA microarrays have proven to be a powerful tool for profiling the expression levels of many genes in a single experiment [11,12]. In addition, several different approaches using microarrays to monitor splicing events have been used successfully [7,13-17]. Probes that span exon-exon junctions can discriminate between isoforms and provide useful information about how exons are joined together. However, since these arrays are typically designed based on observed sequences, junction microarrays are not optimal for discovery of novel isoforms. Several recent studies have shown that a much larger than anticipated fraction of the human genome is transcribed [18-21]. Therefore, it is likely that the current set of annotated exons is incomplete.

In order to create a tool with a higher likelihood of discovering previously unknown transcript variation and content, we have designed a high density oligonucleotide microarray that contains probes for every predicted exon in the human genome. This design uses a combination of gene annotations, sequence information, and gene prediction algorithms as content sources. The design includes four probe pairs (perfect match and single base-pair mismatch) for more than one million predicted exon clusters (groups of overlapping exons). More than 9.6 million unique probes were manufactured onto a 4 chip set using a light-directed *in situ *DNA synthesis approach [22,23]. In order to evaluate transcript variation broadly across the genome and the human body, we hybridized target generated from three biological replicates of sixteen normal adult human tissues, including six sub-regions of the central nervous system.

Using a simple algorithm that normalizes the intensity of each probeset by the overall expression level of the gene the probeset maps to, we identified exons that are specifically enriched in one tissue or a group of tissues. Since the brain is known to be rich in alternative splicing [10,24,25], we focused our analysis on alternatively spliced regions that had statistically different inclusion rates in the six brain tissues as compared to the remaining non-brain tissues. Many of the alternatively spliced regions identified as brain enriched were validated by RT-PCR. In doing so, we identified several new splicing patterns that were not represented in the publicly available sequence data. Furthermore, we discovered several novel exons that demonstrated tissue-specific splicing patterns. The novel exons have probesets on the array due solely to computational prediction and lack support from any cDNA sequence data. Our results suggest that calculations of the frequency of alternative splicing based on cDNA sequence information alone are likely to be an underestimate.

## Results

### Array design

The arrays in the set represent a prototype human exon array and were designed to measure the expression levels of exons as independent objects. The goal of the design was to be as inclusive as possible when selecting content using a combination of exons that have been identified by empirical observation as well as exons derived from computational prediction across the entire human genome. The combination of predicted and confirmed exons will allow us to validate the existence of putative gene content. The ability to treat exons as independent objects provides the opportunity to monitor changes in the patterns of expression at the exon level allowing for detection and quantification of alterations in exon usage and changes in alternative splicing patterns. The design dramatically increases the resolution of whole genome gene expression analysis down to the level of transcript diversity.

**Figure 1:**
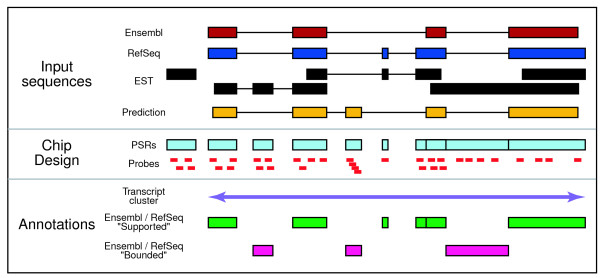
Exon microarray design. Input sequences from a variety of sources were projected onto the November 2002 version of the human genome (hg13). Where possible, up to four probe pairs were selected for each PSR. Probesets that overlap Ensembl or RefSeq sequences are labeled 'Supported.' Probesets that fall within the genomic boundaries of a transcript cluster are labeled 'Bounded.'

The design objective was to include a probeset for every potential exon in the entire human genome. We used a wide variety of predicted and empirically identified transcript/exon collections as input for the probe design algorithms. The collections include Ensembl [26], GenScan [27], Twinscan [28], SLAM [29], as well as direct alignment of cDNA-based content, such as RefSeq mRNAs [30], GenBank mRNAs, and dbESTs. The various input sequences and annotations were consolidated and projected onto the November 2002 (hg13) assembly of the human genome and parsed into unique probe selection regions (PSRs) as defined by the edges of the projections (Figure 1). Across the genome, more than one million exon clusters were identified and grouped into transcript clusters based on overlapping boundaries of input annotations. During the probe selection process, the best four 25-mer probes for expression level analysis were selected from each probe selection region [31]. The median size of all PSRs was 119 base-pairs (bp). Approximately 90% of all PSRs were covered by 4 probe pairs. Any PSR of length greater than or equal to 17 nucleotides (nt) received at least 1 probe pair. Pilot experiments suggested that some signal intensity could be achieved from hybridization to a contiguous perfect match of 17 or more nucleotides. Probes for PSRs that were less than 25 nt were designed with a central target sequence and surrounding genomic sequences filling out the 25-mer. Each probe pair consists of a perfect match probe and a mismatch probe that has a one base substitution in the middle position. The final design included 1.4 million PSRs and more than 9.6 million unique probes that were manufactured onto four 12.8 mm^2 ^arrays with 8 micron feature pitch.

The design was also intended to take alternative 5' or 3' splice sites into consideration. If there was sequence evidence of overlapping exons with different edges (suggesting alternative donor or acceptor sites), then that exon was divided into multiple PSRs. In doing so, the array should be able to detect changes in splice site usage in addition to monitoring inclusion level of the exon as a whole. The PSR size for alternative splice sites is still constrained by the 17 bp minimum. Consequently, splice sites that are shifted by less than 17 nt were not included in the design.

For this design we intentionally omitted exon-exon junction probes. While junction probes provide additional information about the possible splice variants present, they require *a priori *knowledge of those variants. Junction designs are typically dependent on observed junctions and we surmised that even the best enumeration of splice variants to date is substantially incomplete and may introduce an undesired bias into our design. A summary of the chip design details is included in Additional data file 7.

### Determination of detection above background

**Figure 2:**
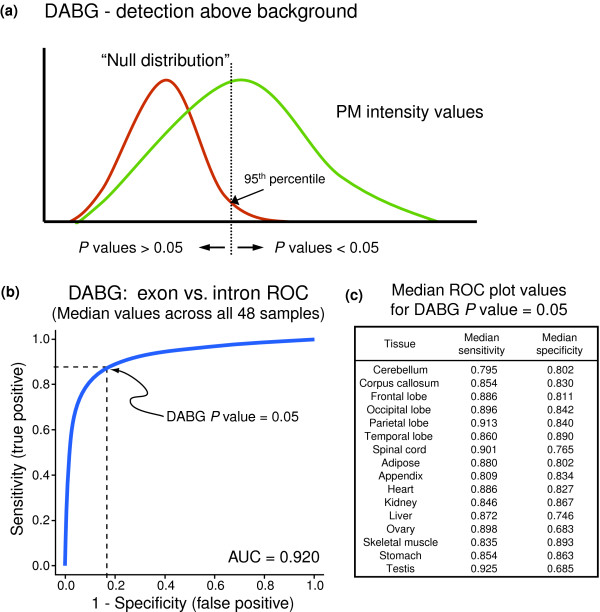
Determination of detection above background. To determine if a given probe signal is detected above background, the PM intensity is compared to a distribution of background probes with the same G/C content. A *p *value is calculated representing the probability that the signal intensity is part of the null distribution. Probes with DABG *p *values of less than 0.05 are considered to be detected above background. For this study, the mismatch probes from PSRs supported solely by GenScan Suboptimal predictions were used to create the null distributions. **(a) **Diagram depicting the comparison of background signal to PM signal. PM signal intensities at the 95% of background probes with the same GC content are given a *p *value of 0.05. **(b) **ROC curve of the median DABG *p *value from all 48 samples. The area under the curve (AUC) is used as a measure of ability of the metric to differentiate between expressed and non-expressed sequences. The sensitivity and specificity values are shown for a DABG *p *value cutoff of 0.05. **(c) **Table of the median sensitivity and specificity values for each tissue using a DABG *p *value cutoff of 0.05.

With only four probes per probe selection region, standard methods for establishing if a particular sequence is present or absent are not effective. As an alternative, we employed a detection metric that compares the intensity from each perfect match probe to a distribution of background probes with similar GC content (Figure 2a). The resulting metric provides a probability that the signal intensity value is part of the background noise (see Materials and methods for details). Thus, probes with low *p *values have signals that are distinct from background and, therefore, considered to be detected above background (DABG).

We employed a receiver operating characteristic (ROC) curve to evaluate the performance of the DABG algorithm. The array design contains exon and intron probesets for a set of 71 housekeeping genes that were previously determined to have constitutive expression across a broad range of tissue samples (see Additional data file 11 for a list of housekeeping genes used). For the purpose of this analysis, we assumed the exons to be present and the introns to be absent. We then evaluated the ability of the DABG metric to separate the control expressed and non-expressed probesets by determining the sensitivity (true positive rate) and specificity (false positive rate) for various DABG *p *value cutoffs. The resulting curve using the median DABG *p *values from all 48 samples is presented in Figure 2b. Using a cutoff *p *value of 0.05, we achieve a median sensitivity of 0.88 (standard deviation (SD) = 0.05) and specificity of 0.82 (SD = 0.07). This value is also nearly optimal for the minimization of type I and type II errors (false positives and false negatives). The median sensitivity and specificity for each of the tissues is shown in Figure 2c.

### Data from tissues

**Figure 3:**
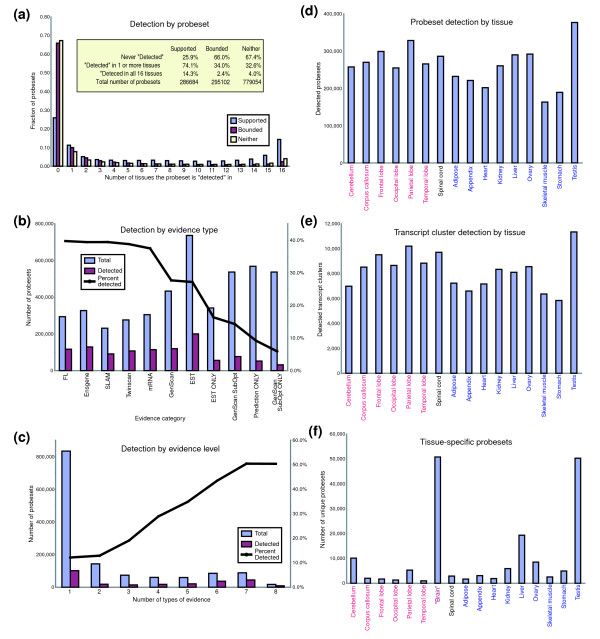
Probeset and transcript cluster detection. **(a) **Bounded, supported, and probesets that fit into neither category are binned by the number of tissues the probeset is detected in. For this and all further analyses, a probeset must have a DABG *p *value less than 0.05 in all 3 biological replicates of a tissue to be considered detected. **(b) **Total number and number of detected probesets listed by evidence type. One probeset may fall into more than one category. Percent detected value is the average detection rate across the 16 tissues. **(c) **Total number and number of detected probesets listed by number of types of evidence. Percent detected value is the average detection rate across the 16 tissues. **(d) **Total number of probesets detected in each tissue. **(e) **Total number of transcript clusters detected in each tissue. For a transcript cluster to be considered detected, a minimum of 50% of Ensembl/RefSeq supported probesets must have DABG *p *values less than 0.05 for at least 2 of 3 replicates. **(f) **Probesets expressed in only one tissue. To be counted a probeset must be detected in all three replicates and not in all three replicates of any other tissue. The 'brain' category represents probesets detected in all three replicates of at least one of the six brain tissues and detected in all three replicates of any non-brain tissues (excluding spinal cord).

To gain a better understanding of the level of transcript variation among normal adult tissues, we generated labeled cDNA target using total RNA from three biological replicates of sixteen different human tissues (see Materials and methods for details). The tissues included cerebellum, corpus callosum, frontal lobe, occipital lobe, parietal lobe, temporal lobe, spinal cord, adipose, appendix, heart, kidney, liver, ovary, skeletal muscle, stomach, and testis. Additional information on the human tissue RNAs is included in Additional data file 8. The majority of Ensembl/RefSeq supported probesets (74.1%) are detected above background (DABG *p *value < 0.05; see Materials and methods for details) in at least one of the 16 tissues. Interestingly, Figure 3a shows that approximately one-third of probesets outside of well annotated exons (bounded) or outside of annotated genes (neither) are detected in at least one of the sixteen tissues. This supports conclusions by others [20,21] that there is a large amount of transcription that occurs outside of annotated genes and also suggests that there is a significant amount of transcript diversity that is missed in the set of curated genes.

Figure 3b shows that in an average tissue, approximately 40% of probesets supported by mRNA sequences or the curated Ensgene set are detected. Gene prediction algorithms that incorporate homology to the mouse genome also have a high rate of detected probesets. Interestingly, nearly 10% of probesets (more than 52,000 per tissue) that are supported only by the prediction algorithms (Genscan, Genscan Suboptimal, Twinscan, SLAM) are detected in an average tissue. The total fraction of probesets present in at least one of the tissues is likely to be higher. The vast majority of probesets (approximately 833,000 of 1,360,000) are supported by only one type of evidence. The inclusive nature of the design results in the majority of probesets being designed to detect speculative content, sequences that do not overlap a well annotated exon. As shown in Figure 3c, of these only 12% are detected, on average. Not surprisingly, the average percent detected number increases as you increase the number of types of evidence per probesets to a maximum of just over 50% for 7 or more types of support evidence (Figure 3c).

There is a fair amount of variability in the total number of probesets detected in each of the tissues. Skeletal muscle, stomach, and heart express the smallest number of probesets, while testis, ovary, liver, and several brain tissues express the largest number (Figure 3d). As expected, the patterns are very much the same when you look at number of transcript clusters (genes) expressed (Figure 3e). With more than 1.36 million probesets on the array, the total number of probesets considered to be detected above background by chance is large. At a *p *value of 0.05, the expected number of false positives with 1.36 million measurements is approximately 68,000. However, when you require the probeset to be detected in all 3 replicates of a tissue, the chances of random detection is dramatically reduced, with only about 170 expected false positives.

In Figure 3f, testis and brain are shown to have a large number of probesets that are tissue-specific; probesets expressed in that tissue and not in any other tissue. These tissue-specific probesets may be from genes that are only expressed in a single tissue, or individual exons that are included in a tissue-specific fashion via alternative splicing. This result is consistent with studies using EST sequences to predict tissue-specific splicing events. Yeo and colleagues [10] found that brain, liver, and testis had the highest number of alternative splicing events using a method that normalizes the number of observed alternative splicing events to the EST coverage in each tissue. Because the various sub-regions of the brain tend to have highly similar splicing and expression patterns, we grouped all of the brain tissues together to identify probesets that are unique to the brain as a whole. This is likely to be an over-simplification and we still expect differences in expression and splicing patterns of the various sub-regions. However, grouping the brain tissues together increases the statistical power to identify genes and individual exons that are enriched in the brain as a whole. Previous studies have shown that the brain itself is very different from other tissues and rich in transcript diversity [10,24,25]. It should be pointed out that the RNA samples are extracted from gross tissue dissections and are likely to contain a mixture of distinct cell types. Expression and splicing patterns are likely to be different between cell types and possibly from cell to cell within the same type. All of this diversity becomes part of the biological noise in the experiment. Our approach should allow us to discover gross relative exon-level changes that are significantly different between one or more tissues.

### Identification of tissue-specific exons using the Splicing Index

**Figure 4:**
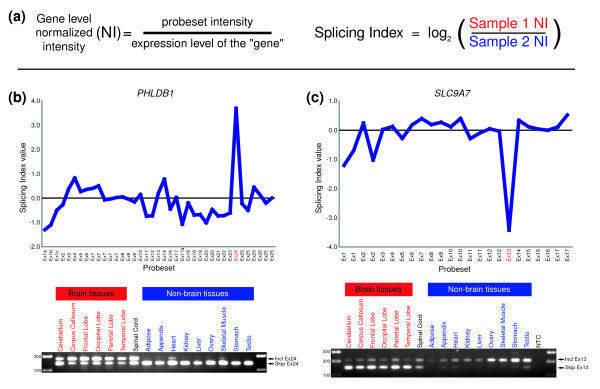
Splicing Index. **(a) **Splicing Index equations. **(b) **PHLDB1: brain-specific Splicing Index values graphed for all Ensembl/RefSeq supported probesets and RT-PCR validation using primers in flanking exons. **(c) **SLC9A7: graphed as in (b).

The array design essentially considers individual exons or parts of exons as independent elements. In theory, this enables a finer resolution view of the transcriptome, using the exon as the fundamental unit rather than the gene. However, it is not possible to use probeset intensities by themselves to find alternatively spliced exons. This is because an alteration in the overall transcription or decay rate of a gene will cause the intensity of all expressed probesets belonging to that gene to change. Ultimately, the goal is to find changes in exon inclusion level that are relative to the expression of the gene. The concept is similar to the indexes used previously to determine splicing efficiency in yeast [13]. By dividing the intensity value of a probeset by an estimate of the expression level of the transcript cluster that the probeset belongs to (Figure 4a), you create a gene-level-normalized intensity that can be compared between samples. Changes in this value between tissues provide a quantitative measure of exon level relative to gene level. Alterations signify changes in exon inclusion rate that may be due to alternative splicing. We refer to comparisons of gene-level-normalized exon expression values as the 'Splicing Index' [32]. A similar approach used in conjunction with a custom exon-junction microarray successfully identified a number of splicing events implicated in breast cancer [33].

Because a consistent gene-level estimate is important to the Splicing Index algorithm, we tested the robustness of the gene-level estimation method to changes in alternative splicing. We simulated exon skipping events by systematically substituting the intensity value of probesets used in the gene-level estimate with the background intensity and re-calculating the gene-level estimate. We then determined the median percent deviation from the original gene-level estimate for every transcript cluster in each of the tissue samples. Using the median intensity of all Ensembl/RefSeq supported probesets as the gene-level estimate proved to be fairly robust to our simulated alternative splicing. Transcript clusters with increased numbers of probesets used in the gene-level estimation tended to have smaller percent deviations. For transcript clusters with 5 or more probesets, 80% of the measurements had median deviations of 26% or less. The median deviations were 18% and 11%, respectively, for transcript clusters with 10 or more and 20 or more probesets used in the gene-level estimation. These data are presented in Additional data file 6.

Exons that are enriched or depleted in one tissue (or group of tissues) compared to another can be identified by looking for a divergence in the gene-level-normalized probeset intensity values. For instance, the brain-specific Splicing Index is calculated by taking the log ratio (base 2) of the median gene-level-normalized intensity of the 18 brain samples (6 tissues, 3 replicates each) and the median gene-level-normalized intensity of the 27 non-brain samples (9 tissues, 3 replicates each). The equation is shown in Figure 4a. The spinal cord samples were excluded from the analysis because previous data had shown spinal cord to have a mixture of brain and non-brain splicing patterns. This value can be calculated for every probeset that maps to or within a transcript cluster.

Two examples are shown in Figure 4b,c. A brain-specific Splicing Index value of 0 indicates that the PSR is present at equal levels in both the brain and non-brain tissues. A positive value implies elevated exon inclusion in brain, and a negative value suggests increased skipping of the exon in brain. In Figure 4b, the majority of exons in the *PHLDB1 *gene are present in both brain and non-brain tissues at roughly equal levels. The spike in index value for the PSR representing exon 24 suggests that this exon is enriched in brain, with an average inclusion rate more than 10-fold higher in the brain tissues. RT-PCR validates the brain-specific inclusion of exon 24 (Figure 4b). The opposite is true for the *SLC9A7 *gene in Figure 4c. Inclusion of the region representing exon 13 is significantly lower in the brain tissues relative to non-brain while most other PSRs have approximately equivalent inclusion levels. Again, RT-PCR verifies that skipping of exon 13 is prevalent in the six brain tissues (Figure 4c).

We used the Splicing Index to identify PSRs that are enriched in the brain. To find statistically significant probesets we carried out a Student *t*-test using the gene-level-normalized probeset intensities comparing the brain tissues as a group to the non-brain tissues. Genes or probesets that have low expression level and, therefore, signal intensities that are in the noise can have a confounding effect on any large-scale automated analysis. Thus, we filtered our results to exclude genes and/or probesets that are not expressed. In order for a transcript cluster to be included in the analysis, we required that at least 50% of the well annotated exons (Ensembl/RefSeq supported probesets) were detected above background (DABG *p *value < 0.05) in more than half of the samples in each group. In addition, we required that any single probeset be detected (DABG *p *value < 0.05) in more than one half of the samples in at least one of the two groups. We used the Benjamini-Hochberg method of multiple testing correction [34,35]. Utilizing a false discovery rate of 0.05 resulted in a *p *value cut-off of 0.012.

While critical for discovery of novel splicing events, the large number of speculative probesets on the array necessitates careful filtering to reduce potential false positives. In the interest of enriching the dataset for true positives, the Splicing Index results were further filtered to remove probesets with the highest likelihood of introducing false positives. Probesets that mapped to multiple genomic locations were discarded (approximately 6.8% of probesets). Probesets with intensity values that were highly discordant with the gene expression level (probesets with intensities greater than ten-fold higher than the median intensity of Ensembl/RefSeq supported probesets) were likewise rejected. In addition, genes with greater than ten-fold changes in average expression level between the brain and non-brain tissue groups were also removed. Despite the fact that the Splicing Index approach is designed to mitigate differences in gene expression level, previous experience has shown that very large differences in gene expression level can amplify the noise leading to potential false positives. After filtering, 10,148 probesets with statistically significant differences in gene-level normalized intensities between the brain and non-brain tissues remained. Of these, 2,161 are indicated as being enriched in the brain tissues relative to non-brain tissues. These 2,161 probesets from 1,097 transcript clusters (genes) represent potential differentially alternatively spliced regions.

### Experimental validation of predicted brain-enriched exons

**Figure 5:**
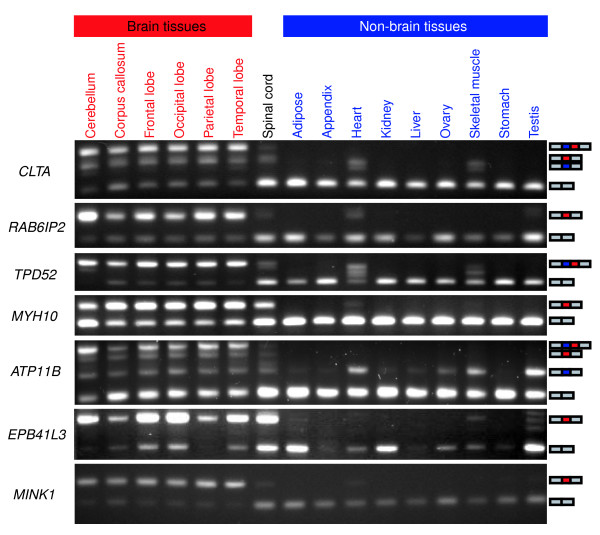
RT-PCR validation of brain-enriched exons identified by the Splicing Index algorithm. Approximately 15 μl of PCR product were separated on a 2.5% agarose gel stained with ethidium bromide. Primers are designed to well annotated exons that flank the PSR identified as brain enriched by the Splicing Index. Primer sequences are available in Additional data file 9.

RT-PCR validation of the increased inclusion in the brain tissues of several of the identified probesets is shown in Figure 5. In order to evaluate the performance of the Splicing Index algorithm for identifying differentially regulated, tissue-specific splicing events from the exon array data, we systematically tested the top hits sorted either by *t*-test *p *value or magnitude of change. For simplicity, we focused our validation efforts on internal alternative splicing events that could be tested using primers in constitutive exons flanking the identified probeset. Therefore, probesets at the extreme edges of transcript clusters and probesets mapping to annotated alternative transcriptional start sites, alternative 3' terminal exons, or alternative polyadenylation regions were not tested. Finally, the Splicing Index results were manually filtered by BLATing [36] the probeset sequence to the UCSC Genome Browser [37]. By observing the probeset location in genomic context we were able to remove probesets with ambiguous transcript cluster assignment and probesets within regions of overlapping transcription units.

After filtering, the 32 probesets with the most significant *p *values were tested by RT-PCR. Of the 32 probesets, 27 (84.4%) showed clear enrichment in the brain tissues by RT-PCR using primers in flanking exons. The higher molecular weight band present in the six brain tissues indicates inclusion of additional sequence in the mRNAs expressed in those tissues via alternative splicing. In nearly all cases, the size difference between the skip and include products is identical to the size of the exon that the identified probeset was designed to detect. A commonly observed artifact for RT-PCR products of alternatively spliced regions is visible on several of the gels. This intermediately sized band typically migrating just below the larger of the two isoforms represents a heteroduplex of the two alternatively spliced forms [38,39]. All RT-PCR gels not present in Figure 5 are shown in Additional data file 4. We also carried out RT-PCR on the probesets with the largest magnitude change of gene-level normalized intensity. Of the top 23, 21 (91.3%) probesets demonstrated clear patterns of brain-specific alternative splicing. Altogether, a total of 84 predicted tissue-enriched probesets identified by the Splicing Index have been evaluated in the course of this project with a 86% (72/84) validation rate.

### Discovery of novel brain-enriched exons

Among the 2,161 probesets identified as brain-enriched by the Splicing Index, 287 of them were supported only by exon predictions (Genscan, Genscan Suboptimal, Twinscan, SLAM). Inclusion of these exons in transcripts produced in the brain would represent novel splicing events since neither the exons themselves nor the splicing events involving the exons have been observed in publicly available cDNA sequences. Several potential novel brain-enriched exons were selected and inclusion of these exons in the brain was verified by cloning and sequencing RT-PCR products from one or more brain tissues. RT-PCR using primers in known exons that surround the predicted exons for several of these genes is shown in Figure 5.

**Figure 6:**
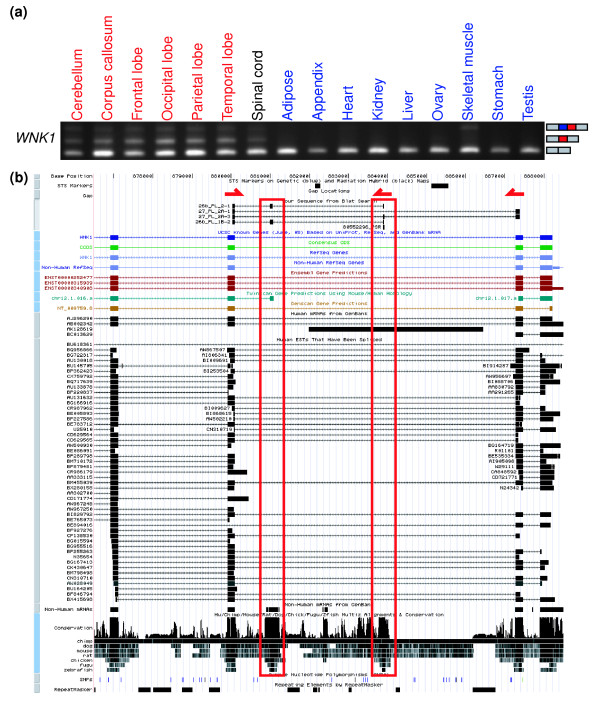
Discovery of novel exons. **(a) **RT-PCR validation of predicted brain-enriched exons. **(b) **Sequences of RT-PCR products from parietal lobe RNA aligned to the human genome using the BLAT tool [36] available on the UCSC Genome Browser website [53].

In Figure 6, the sequences of RT-PCR products for the *WNK1 *gene shows the inclusion of two novel exons in the brain tissues. The sequences were BLATed [36] to the human genome and displayed in the UCSC Human Genome Browser [37]. The newly identified exons do not overlap any mRNA or EST sequences. Probesets were designed because the exons were predicted from one of the *in silico *gene finding algorithms. As can been seen near the bottom of Figure 6b in the conservation tract, the sequences of the exons themselves are well conserved in a variety of other organisms. As has been seen for many regulated alternatively spliced exons, the sequence similarity extends into the surrounding intron, suggesting possible conservation of a shared regulatory element [40-42]. This also highlights the value of sequence conservation in identifying exons and regulatory motifs using sequence information alone.

Altogether, our initial, exploratory analysis revealed 17 novel exons in 11 genes. In addition, six new alternative splicing patterns involving annotated exons were discovered during the RT-PCR validation process. These six new splicing events include four instances of novel arrangements of annotated exons, one new alternative 5' splice site and one new alternative 3' splice site (see Additional data file 9 for details). The sequences of the novel exons and new alternative splicing events have been deposited in GenBank (GenBank:DQ925667-DQ925693 and GenBank:EF139845-EF139860). Information on the novel exons, including RT-PCR primer sequences can be found in Additional data file 9. In addition, RT-PCR gels for all other novel exons not included in Figure 5 are included in Additional data files 1 and 4. These data demonstrate the ability of an inclusive exon array design to discover novel exons and novel alternative splicing events. It also suggests that the catalog of splicing events in publicly available sequences is far from complete.

### Estimates of the frequency of alternative splicing

To estimate the frequency of alternative splicing among our set of normal adult human tissues, we compared each tissue to every other tissue. In all, 120 pair-wise comparisons were carried out among the 16 tissues. Gene-level normalized intensities were calculated for each probeset that fell within the bounds of a transcript cluster. The values from three biological replicates of one tissue were compared to the three biological replicates of another tissue using a Student *t*-test. To reduce the number of false positives, we filtered out non-expressed genes by requiring that a given transcript cluster be 'detected' in at least two of the three replicates in each tissue. A gene is considered present if more than half of the Refseq/Ensembl supported probesets were detected above background with a *p *value of 0.05 or less. In addition, each probeset must be detected above background (DABG *p *value ≤ 0.05) to be included in that particular pair-wise comparison. Filtering steps similar to those used in the selection of brain-enriched PSRs were also employed to remove potential cross-hybridizing probesets and genes with greater than ten-fold differences in gene-expression level. It should be noted that for alternative splicing to be detected by the Splicing Index algorithm, the splicing pattern must change between tissues. For example, a gene that expresses multiple isoforms in the same proportion in each tissue tested will not be detected as differential alternative splicing.

A total of 19,221 transcript clusters ('genes') were used as input to the analysis. Of these, 12,139 were detected in a minimum of two tissues. There were 8,837 transcript clusters that had at least one probeset with a Splicing Index *t*-test *p *value of less than 0.001 and minimum magnitude of change greater than 0.5, suggesting differential exon usage in at least one comparison. This means that 72.8% (8,837/12,139) of detected genes displayed evidence of differential alternative splicing among the 16 normal adult human tissues. Additional data file 2 contains details on the number of probesets identified as differentially expressed in each of the pair-wise tissue comparisons.

In an effort to demonstrate the validity of the pair-wise comparison approach, we calculated the frequency that the RT-PCR validated brain-enriched probesets were identified in comparisons of a single brain tissue (three biological replicates) with a single non-brain tissue (three biological replicates). For each probeset, there were a total of 54 possible 'brain versus non-brain' comparisons, 36 possible 'non-brain versus non-brain' comparisons and 15 possible 'brain versus brain' comparisons considered in this analysis (comparisons involving spinal cord were excluded from the analysis). Using the same criteria as above, if the gene was not detected in both tissues, that particular comparison was excluded from consideration. Employing a minimum fold change of 0.5 and *t*-test *p *value of less than 0.01, the median frequency of identification in single brain tissues versus one non-brain tissue was 62.7%. This is relative to a median frequency of 11.1% of single non-brain tissues compared to another single non-brain tissue and a median frequency of 0% of a single brain tissue compared to another single brain tissue. Because many of the identified brain-enriched exons are not completely unique to the brain, we did not expect to identify our validated brain-enriched probesets in 100% of the 'brain versus non-brain' comparisons. These results demonstrate that the single tissue pair-wise comparison approach can detect the majority of validated brain-enriched probesets. In addition, the data verifies the brain-enrichment of these probesets and suggests that the set of splicing patterns of these identified exons is fairly consistent across multiple sub-regions of the central nervous system.

The RT-PCR validation focused primarily on cassette exons, but the exon microarray is also designed to detect the usage of alternative 5' and 3' splice sites. Thus, to get a sense of the frequency of alternative splice site usage we calculated the Pearson correlation coefficient of signal intensities across the 16 tissues for probesets belonging to the same exon cluster. A total of 277,838 (26.9%) exon clusters have more than one probeset. For simplicity, our analysis considered only Ensembl/RefSeq supported probesets that were expressed in more than 50% of the tissues. Probesets from the same exon cluster generally had very high correlation with a median of 0.83. This is relative to a median correlation of -0.01 for probesets selected randomly from 2 different exon clusters (Additional data file 5). Approximately 21% of exon clusters had correlation values less than 0.5. While this is not a direct measure of the frequency of alternative 5' and 3' splice site usage, it does provide a sense of how often probesets from the same exon cluster are divergent, which may be due to alternative splice site usage.

## Discussion

Due to the inclusive nature of our genome-wide exon microarrays, we are able to discover novel alternative splicing patterns in addition to monitoring known splicing events. As technology allows for smaller feature sizes, it will be possible to include larger numbers of probes per array, thereby increasing the feasibility of large-scale non-biased designs. Microarray designs that are more inclusive and less dependent on current genome annotations will ultimately aid in the development of tools that are designed to annotate, rather than the other way around. For example, recent work using a similar design concept based on the mouse genome was able to refine gene boundaries using co-regulation of exons across many different tissue types [43]. However, our data suggest that there is a large amount of expression outside of well annotated exons. Reliance on any one single exon prediction algorithm or sequence content source will likely result in incomplete coverage.

Exon microarrays represent a huge step forward in the resolution level of gene study. The tool makes it possible to use the entire genome as a splicing reporter. Future studies using this technology should aid in the discovery of splicing regulatory mechanisms and expand our knowledge of how alternative splicing is controlled. In addition to splicing information, the exon array format provides excellent coverage at the gene level. With more than 40 individual probes for the average gene, robust gene-level expression estimates are likely to be more sensitive than array designs with fewer probes per gene.

We have demonstrated the use of a simple and easy to implement algorithm for finding changes in alternative splicing patterns from exon array data. While there are more sophisticated techniques for finding splicing patterns from microarray data [16,44,45], the Splicing Index [32] was intended to be a straightforward example of a method for identifying tissue-specific exons. The method combined with standard statistical tests is robust and was able to identify known brain-specific splicing patterns. In addition, we discovered novel exons involved in alternative splicing events regulated in a brain-specific pattern. Our data suggest that a significant proportion of isoforms is yet to be discovered and may indicate that the frequency of alternative splicing based on cDNA sequences is an underestimate. In fact, pair-wise comparisons of the 16 tissues suggest that nearly three-quarters of genes are differentially alternatively spliced. This value is very close to other estimates of the frequency of alternative splicing based on microarray experiments [7]. However, our estimation is potentially an underestimate of the total amount of alternative splicing since our tissue set is limited and did not include any samples from fetal or diseased tissues.

By using a strict set of filtering criteria and by employing a manual filter by which the data for potential validation targets were observed in a genomic context, we were able to limit the number of false positives. The RT-PCR validation rate for all splicing events examined in this study was approximately 86%. Many of the validation targets mapped to exons that were previously known to be alternatively spliced based on sequence information from the UCSC Genome Browser and validation rates for probesets mapping to these events was the highest. Approximately 98% (46/47) of annotated alternative splicing events highlighted by the Splicing Index showed clear enrichment of the queried exon by RT-PCR. This situation is a more appropriate comparison to many exon-junction arrays since the designs are already filtered for known splicing events [14,33,45-47]. The validation rate for the purely novel exons was considerably lower, with 11 of 16 targets (69%) validated by RT-PCR. A negative RT-PCR result, however, does not necessarily mean that the identified probeset is a false positive. It only signifies that the exon is not included in a transcript that contains the exons to which the primers were designed. The sequence may still be expressed as an independent transcript or in a splice variant that does not include the flanking primer exons. This may be more likely in the case of potential novel exons that are only predictions or have minimal sequence support since designing primers for these sequences is problematic. Probesets with splicing index *t*-test *p *values as high as 2.5 × 10^-4 ^and splicing index values as low as 0.6 still gave positive validation results by RT-PCR. In many cases, the exons overlapping those probesets had less dramatic splicing pattern changes or identified exons that were not completely unique to brain. The high validation rate clearly demonstrates the ability of the Splicing Index algorithm to identify exons that are significantly enriched in one tissue or a group of tissues relative to another.

While microarray designs that include exon-exon junctions do provide an additional bit of information about how exons are joined together, the design is not optimal for discovery of new splicing events. Johnson and colleagues [7] were able to detect new exon skipping events by utilizing a human exon junction microarray. However, their approach is not capable of detecting new splicing events involving novel exons since their microarray was designed solely on observed junctions in RefSeq mRNA sequences, which do not comprise a complete enumeration of splice variants. Exon and junction type array designs each have their pros and cons. A combination of both approaches may provide additional power in the study of alternative splicing. Even our set of predicted exons is likely to be incomplete. Small exons that are less than 17 bp, for example, are not included in our design. It is also possible for an exon to be missed if it was not predicted by any of the design inputs or if the PSR sequence itself was not amenable to designing an acceptable probe. The design was, however, intended to be as inclusive as possible. It represents an improvement of more than an order of magnitude in genome coverage over currently available tools.

To date, most microarray-based target discovery programs in the pharmaceutical industry have been limited to looking at a single version of a transcript. We did a cursory analysis of the number of potential drug targets that exhibit potential tissue-specific alternative splicing patterns. Nearly all small-molecule drug targets fall into 130 InterPro domain protein families [48]; 3,365 genes (transcript clusters) containing one of these protein domains were identified. These 3,365 genes nominally represent the 'drugable genome.' Approximately 77% of detected genes from this group show evidence of alternative splicing in our pair-wise comparison of 16 normal adult human tissues. Additional data file 3 shows that different classes of drugable genes display variable amounts of differential alternative splicing. For example, the data suggest that nearly 85% of detected serine/threonine protein kinase genes may be differentially alternatively spliced. The largest class of genes is the Rhodopsin-like GPCR with more than 600 members. Likely due to their low expression levels, only about 150 of these genes are detected among the 16 tissues. And of those, only about a third present evidence of differential alternative splicing (Additional data file 3). Overall, the high percentage of drugable genes with potential tissue-specific isoforms may represent an untapped source of information that could benefit drug discovery programs.

Using the exon microarray technology, we will be able to measure changes in the splicing patterns of thousands of genes at once and discover new patterns of alternative splicing. Given the diversity of transcripts likely to be created from genes, and the importance of detecting and differentiating these transcripts for the correct interpretation of biological systems, we believe our arrays are a useful, enabling tool for further study. 'Gene expression' is properly becoming 'transcript expression.'

## Conclusion

In this work we analyzed the expression of more than one million individual annotated and predicted exons across three biological replicates of sixteen normal adult human tissues using a prototype exon microarray. Our data suggest significant expression from regions outside of well annotated exons and well annotated genes, with nearly one-third of probesets in these regions detected above background in the average tissue. Using a conceptually simple algorithm called the Splicing Index that normalizes exon intensity by expression level of the gene, we identified and validated a number of exons that were enriched in samples from six sub-regions of the brain relative to nine non-brain tissues. After rational filtering of the Splicing Index results, systematic RT-PCR examination of the top scoring hits resulted in a validation rate of around 86%. Pair-wise comparisons of each of the 16 tissues suggests that approximately 73% of expressed genes are potentially differentially alternatively spliced. In addition, we demonstrate how the inclusive nature of the exon array can identify novel exons that display tissue-specific splicing patterns. Our cursory examination validated 17 such novel exons in 11 genes. An additional six examples of new alternative splicing patterns involving annotated exons were also discovered. The analysis methods presented here are applicable to a wide range of microarray designs including exon, junction, and tiling arrays. The exon level data generated from the 16 tissues will likely be a rich source of information for the study of the regulation of alternative splicing.

## Materials and methods

### Target preparation and hybridization

Total RNA was purchased from Biochain (Hayward, CA, USA). RNA samples came from age matched normal human males (excluding female-specific organs) with three biological replicates for each of the 16 tissues. Total RNA (100-200 ng) was reverse transcribed using a random hexamer/T7-promoter oligonucleotide (5'-GAATTGTAATACGACTCACTATAGGGNNNNNN-3') for initial strand priming. Second strand cDNA was generated using Klenow Fragment (NEB; Ipswich, MA, USA) and amplified into cRNA by T7 RNA Polymerase-based IVT (MEGAscript T7, Ambion; Austin, TX, USA). Resulting cRNA was cleaned up on an RNeasy column (Qiagen; Valencia, CA, USA). cRNA was converted back into cDNA via reverse transcription with random hexamers. The second strand was generated and cDNA was cleaned up on a QIAquick PCR Purification column (Qiagen). Double-stranded cDNA was fragmented with 0.6 U DNaseI (Promega; Madison, WI, USA) at 37°C for 10 minutes. Fragment size was checked using a Bioanalyzer (Agilent; Santa Clara, CA, USA) with optimal fragment size in the 50-200 bp range. Fragmented cDNA was end labeled with a biotin-conjugated nucleotide analog (DLR-1a; Affymetrix, Inc.) using terminal transferase (Roche; Nutley, NJ, USA). Fragmented and labeled cDNA was hybridized for 16 hours at 50°C in a hybridization solution containing 7% DMSO. A constant mass of 20 μg of double-stranded cDNA was hybridized to each array. One hybridization mixture per sample was hybridized serially to the four chip set over four consecutive days. After hybridization, arrays were stained and washed on a GeneChip Fluidics Station (Affymetrix, Inc.) using standard protocols for eukaryotic arrays. Additional information on the performance of the assay can be found online [49].

### Data acquisition and analysis

Following staining and washing, the arrays were scanned using a GeneChip Scanner 3000 (Affymetrix, Inc.). The 8 micron pitch features on the array were scanned at 1.09 micron pixel resolution and probe intensity is reported after image alignment and gridding using GeneChip Operating System software (Affymetrix, Inc.). Arrays were normalized using individual probes from 675 probesets from 71 empirically derived housekeeping genes shown to be consistently expressed across many tissues. The median perfect match (PM) value for this collection of normalization controls is set to 700 and is used to calculate a scaling factor which is then applied to all PM and mismatch (MM) probes. These probes are present on all four chips in the array set. Probeset intensities were generated from individual PM and single base MM probes using the simplified expression analysis (SEA) method. In short, PM intensities are attenuated to reflect the background as measured by the MM probes. The SEA probeset value is the median probe intensity using the following formula:

U(i,j) = ((pm - mm) + sqrt((pm - mm)^2 + 4 × L × pm × mm))/2

We used a value of 0.005 for L, the attenuation term. Additional information about the SEA algorithm can be found online [50]. Microarray design information and array data are available through the NCBI Gene Expression Omnibus (series accession number GSE5791) [51].

### Determination of detection above background

To determine if the signal intensity for a given probe is above the expected level of background noise, we compared the signal for each probe to a distribution of signals from probes with the same GC content that are not expected to detect real target. For this study, we used the single base mismatch probes from PSRs that were supported only by Genscan Suboptimal predictions. This represented a large group of probes that were the least likely to detect real target and, therefore, likely to reflect background signal. We generated a *p *value representing the probability that the signal intensity of a given probe is part of the background distribution (Additional data file 4). The *p *value is determined by comparing the PM intensity for a given probe to the intensity for the group of background probes with the same GC content. The probes are ranked by intensity and the *p *value is calculated by dividing the rank of the probe by the total number of background probes in that GC bin. Thus, a probe *p *value of 0.05 indicates that the PM intensity is larger than the 95% percentile of the background probes with the same GC content. Probes with small *p *values had signal that was separable from noise and, therefore, considered to be DABG. The individual *p *values from all probes in a probeset were combined using Fisher's Exact test to generate a *p *value for the probeset. A probeset that had a DABG *p *value of 0.05 or less was considered to be detected (above background).

### RT-PCR and sequence validation

Approximately 2 μg of total RNA was reverse transcribed using the TaqMan Reverse Transcription Reagents kit per the manufacturer's instructions (Applied Biosystems; Foster City, CA, USA). A combination of oligo dT and random hexamers were used to prime reverse transcription. PCR primers were designed in constitutive exons flanking the target sequence. PCR was carried out using Taq Polymerase (Promega) per the manufacturer's instructions using approximately 50-100 ng of cDNA as template. PCR products were separated on 2.5% agarose gels and stained with ethidium bromide for visualization. Selected RT-PCR products were gel purified and cloned into a TA Cloning vector (Invitrogen; Carlsbad, CA, USA) for sequencing. All RT-PCR primer sequences are included in Additional data file 9.

## Additional data files

The following additional data are available with the online version of this paper. Additional data file 1 provides RT-PCR results of novel tissue-specific exons. Approximately 15 μl of PCR product were separated on a 2.5% agarose gel stained with ethidium bromide. Primers were designed to well annotated exons that flank the PSR identified as a potential novel tissue-specific exon by the Splicing Index. Primer sequences are available in Additional data file 9. Additional data file 2 includes pair-wise tissue Splicing Index results. The three biological replicates of each individual tissue were compared to each of the other tissues using the Splicing Index algorithm. The Splicing Index compares gene-level normalized probeset intensities using a Student *t*-test. **(a) **The number of probesets that have significantly different inclusion rates between the two tissues. Probesets with *p *values less than 0.05 were considered significant. **(b) **The number of significantly different probesets normalized by the number of genes used in that comparison. In order for a gene to be included in each of the pair-wise comparisons, 50% of the Ensembl/RefSeq supported exons were required to have DABG *p *values less than 0.05 in a minimum of two out of the three biological replicates of each tissue in that comparison. In all, 9085 of 12,139 genes (74.8%) showed evidence of differential alternative splicing in at least one tissue comparison. Additional data file 3 shows alternative splicing in the drugable genome. The number of genes, number of detected genes, and number of genes displaying differential exon expression is graphed for several of the largest classes of drugable genes. Gene classes are sorted from left to right by the percentage of genes exhibiting differential alternative splicing. Additional data file 4 shows additional RT-PCR of predicted brain-enriched exons. See Additional data file 1 for details. Sequences of the primers used in the RT-PCR are available in Additional data file 10. Additional data file 5 shows the correlation of exon cluster probeset intensities. **(a) **A histogram of the Pearson correlation coefficient of signal intensity across the 16 tissues for probesets belonging to the same exon cluster and probesets randomly selected from different exon clusters. **(b) **A table showing the median and average correlation and the total number and percent of exon clusters with correlations less than different values. Additional data file 6 shows the robustness analysis of gene-level estimation. The robustness of the gene-level estimation method to alternative splicing was analyzed by simulating exon skipping events. The intensity of each probeset that was used in the gene-level estimate was systematically substituted for the background level and the gene-level estimate was re-computed. The deviation of the altered gene-level estimate from the original gene-level estimate was determined for every transcript cluster in each tissue sample. **(a) **The median percent deviation was calculated by taking the median of the difference of the altered and the original gene-level estimates divided by the original gene-level estimate all multiplied by 100. **(b) **A graph illustrating the median percent deviation of gene-level estimates for transcript clusters with 5 or more, 10 or more, and 20 or more probesets used in the calculation of the gene-level estimate. Additional data file 7 lists the chip design information. Additional data file 8 contains RNA sample information. Additional data file 9 lists the RT-PCR primer sequences. Additional data file 10 lists additional RT-PCR primers. Additional data file 11 lists the normalization control genes.

## Supplementary Material

Additional data file 1 Approximately 15 μl of PCR product were separated on a 2.5% agarose gel stained with ethidium bromide. Primers were designed to well annotated exons that flank the PSR identified as a potential novel tissue-specific exon by the Splicing Index. Primer sequences are available in Additional data file 9. Click here for file

Additional data file 2 The three biological replicates of each individual tissue were compared to each of the other tissues using the Splicing Index algorithm. The Splicing Index compares gene-level normalized probeset intensities using a Student *t*-test. **(a) **The number of probesets that have significantly different inclusion rates between the two tissues. Probesets with *p *values less than 0.05 were considered significant. **(b) **The number of significantly different probesets normalized by the number of genes used in that comparison. In order for a gene to be included in each of the pair-wise comparisons, 50% of the Ensembl/RefSeq supported exons were required to have DABG *p *values less than 0.05 in a minimum of two out of the three biological replicates of each tissue in that comparison. In all, 9085 of 12,139 genes (74.8%) showed evidence of differential alternative splicing in at least one tissue comparison. Click here for file

Additional data file 3 The number of genes, number of detected genes, and number of genes displaying differential exon expression is graphed for several of the largest classes of drugable genes. Gene classes are sorted from left to right by the percentage of genes exhibiting differential alternative splicing. Click here for file

Additional data file 4 See Additional data file 1 for details. Sequences of the primers used in the RT-PCR are available in Additional data file 10. Click here for file

Additional data file 5 **(a) **A histogram of the Pearson correlation coefficient of signal intensity across the 16 tissues for probesets belonging to the same exon cluster and probesets randomly selected from different exon clusters. **(b) **A table showing the median and average correlation and the total number and percent of exon clusters with correlations less than different values. Click here for file

Additional data file 6 The robustness of the gene-level estimation method to alternative splicing was analyzed by simulating exon skipping events. The intensity of each probeset that was used in the gene-level estimate was systematically substituted for the background level and the gene-level estimate was re-computed. The deviation of the altered gene-level estimate from the original gene-level estimate was determined for every transcript cluster in each tissue sample. **(a) **The median percent deviation was calculated by taking the median of the difference of the altered and the original gene-level estimates divided by the original gene-level estimate all multiplied by 100. **(b) **A graph illustrating the median percent deviation of gene-level estimates for transcript clusters with 5 or more, 10 or more, and 20 or more probesets used in the calculation of the gene-level estimate. Click here for file

Additional data file 7 Chip design information. Click here for file

Additional data file 8 RNA sample information. Click here for file

Additional data file 9 RT-PCR primer sequences. Click here for file

Additional data file 10 Additional RT-PCR primers. Click here for file

Additional data file 11 Normalization control genes. Click here for file
