# Supplementary figures and images for: Discovery of tissue-specific exons using comprehensive human exon microarrays
Source: Genome Biol. 2007 Apr 24;8(4):R64. doi: 10.1186/gb-2007-8-4-r64 (PMC1896007; doi:10.1186/gb-2007-8-4-r64)

# Additional File 1 - RT-PCR Results of Novel Tissue-Specific Exons

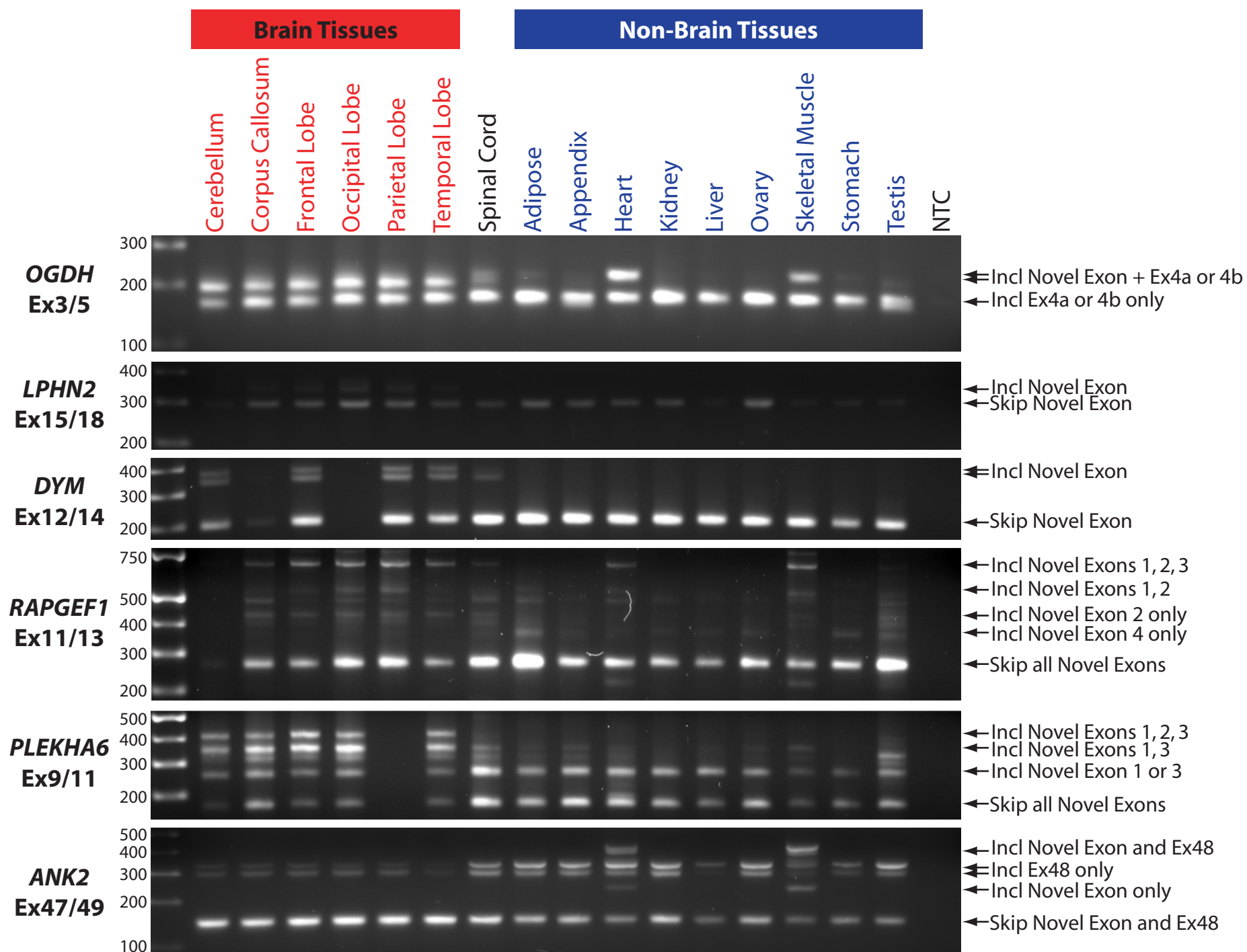

Supplement: Additional data file 1 — Approximately 15 μl of PCR product were separated on a 2.5% agarose gel stained with ethidium bromide. Primers were designed to well annotated exons that flank the PSR identified as a potential novel tissue-specific exon by the Splicing Index. Primer sequences are available in Additional data file 9. [file gb-2007-8-4-r64-S1.pdf]

Additional File 3 - Alternative Splicing in the Drugable Genome

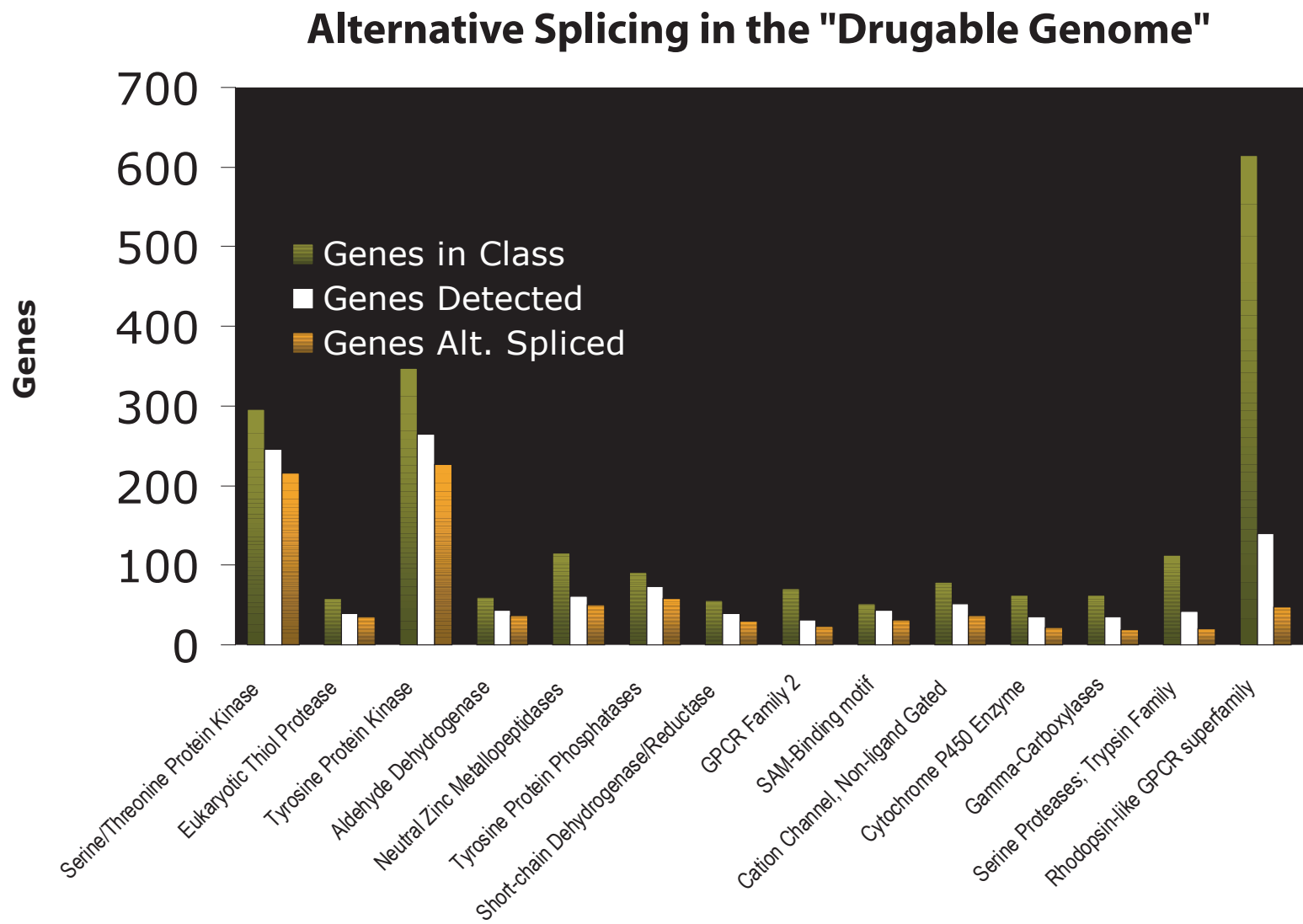

Supplement: Additional data file 3 — The number of genes, number of detected genes, and number of genes displaying differential exon expression is graphed for several of the largest classes of drugable genes. Gene classes are sorted from left to right by the percentage of genes exhibiting differential alternative splicing. [file gb-2007-8-4-r64-S3.pdf]

Additional File 4

Additional RT-PCR Validation of Predicted Brain-Enriched Exons

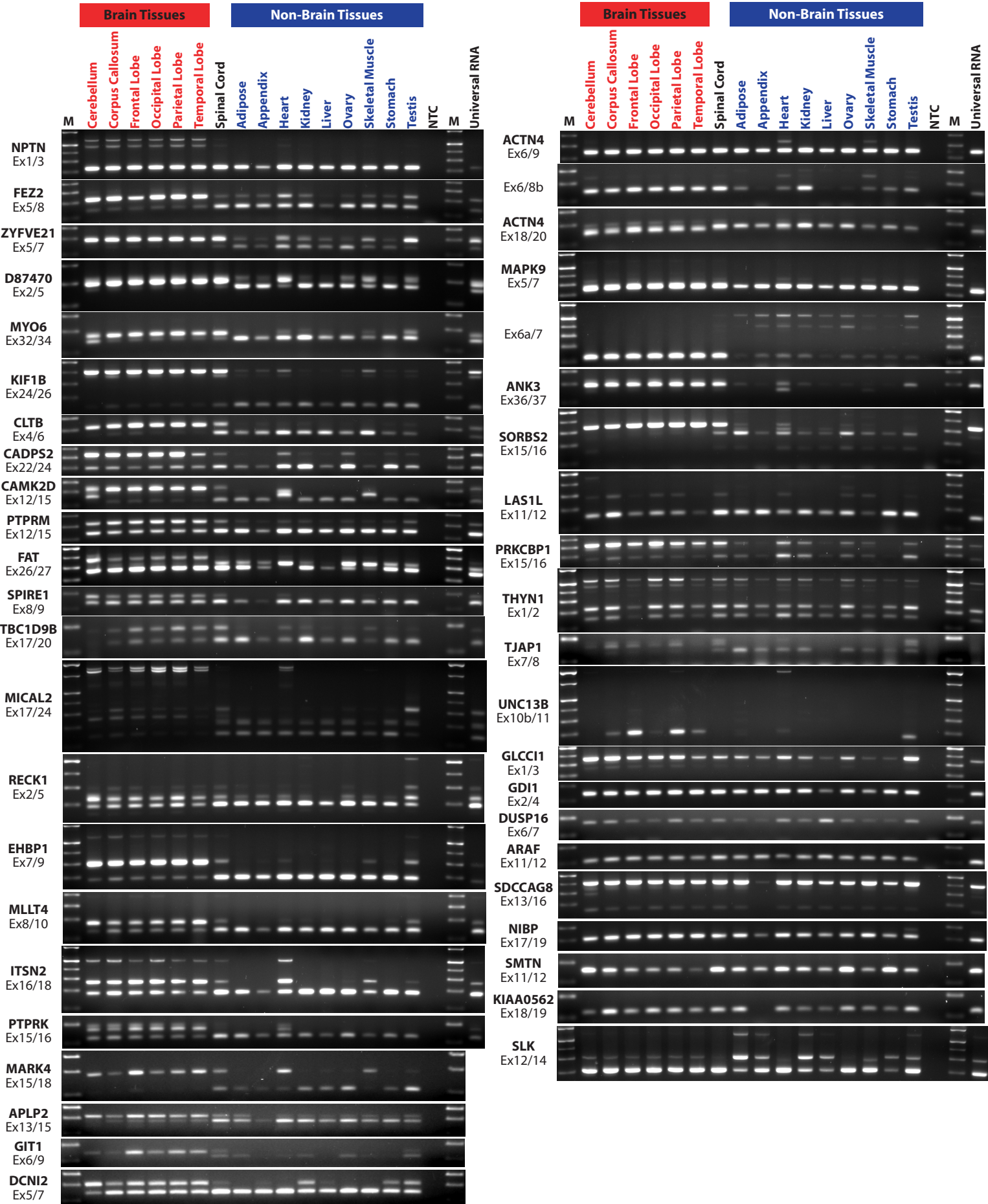

Supplement: Additional data file 4 — See Additional data file 1 for details. Sequences of the primers used in the RT-PCR are available in Additional data file 10. [file gb-2007-8-4-r64-S4.pdf]
