# Supplementary material for: Discovery of tissue-specific exons using comprehensive human exon microarrays
Source: Genome Biol. 2007 Apr 24;8(4):R64. doi: 10.1186/gb-2007-8-4-r64 (PMC1896007; doi:10.1186/gb-2007-8-4-r64)
Supplement: Additional data file 2 — The three biological replicates of each individual tissue were compared to each of the other tissues using the Splicing Index algorithm. The Splicing Index compares gene-level normalized probeset intensities using a Student t-test. (a) The number of probesets that have significantly different inclusion rates between the two tissues. Probesets with p values less than 0.05 were considered significant. (b) The number of significantly different probesets normalized by the number of genes used in that comparison. In order for a gene to be included in each of the pair-wise comparisons, 50% of the Ensembl/RefSeq supported exons were required to have DABG p values less than 0.05 in a minimum of two out of the three biological replicates of each tissue in that comparison. In all, 9085 of 12,139 genes (74.8%) showed evidence of differential alternative splicing in at least one tissue comparison. [file gb-2007-8-4-r64-S2.pdf]

Additional File 2 - Pair-wise Tissue Splicing Index Results

A

Number of Alternatively Spliced Probesets by Comparison

	Cerebellum	Corpus Callosum	Frontal Lobe	Occipital Lobe	Parietal Lobe	Temporal Lobe	Spinal Cord	Adipose	Appendix	Heart	Kidney	Liver	Ovary	Skeletal Muscle	Stomach
Corpus Callosum	1281														
Frontal Lobe	1665	300													
Occipital Lobe	2210	1413	1622												
Parietal Lobe	2793	739	102	2459											
Temporal Lobe	2358	392	246	1925	479										
Spinal Cord	1365	280	598	1595	895	897									
Adipose	903	945	1442	2211	2103	2025	744								
Appendix	442	925	1413	1741	2063	1968	893	129							
Heart	1122	610	852	516	1303	851	603	756	598						
Kidney	1176	1240	1532	2485	2002	2040	979	587	338	779					
Liver	2022	2065	2833	2952	4335	3864	1569	1808	1097	1303	2363				
Ovary	2138	1957	2686	862	3913	3246	1596	2313	1419	397	2416	2315			
Skeletal Muscle	755	852	849	1274	1049	1034	575	353	512	390	478	1419	1449		
Stomach	1335	1123	1395	449	1937	1601	939	1322	899	167	1400	1328	223	731	
Testis	4209	4116	5042	2509	7247	6060	3563	4037	2932	1361	4235	4772	1767	2658	1048

B

Alt. Spliced Probesets Divided by Number of Genes Detected

	Cerebellum	Corpus Callosum	Frontal Lobe	Occipital Lobe	Parietal Lobe	Temporal Lobe	Spinal Cord	Adipose	Appendix	Heart	Kidney	Liver	Ovary	Skeletal Muscle	Stomach
Corpus Callosum	0.22														
Frontal Lobe	0.27	0.04													
Occipital Lobe	0.39	0.20	0.21												
Parietal Lobe	0.45	0.09	0.01	0.31											
Temporal Lobe	0.40	0.05	0.03	0.26	0.06										
Spinal Cord	0.23	0.04	0.07	0.22	0.11	0.12									
Adipose	0.19	0.17	0.25	0.41	0.36	0.37	0.12								
Appendix	0.10	0.18	0.27	0.37	0.39	0.40	0.16	0.03							
Heart	0.25	0.11	0.15	0.09	0.22	0.16	0.10	0.14	0.13						
Kidney	0.23	0.20	0.24	0.43	0.30	0.34	0.15	0.10	0.07	0.14					
Liver	0.42	0.35	0.46	0.52	0.68	0.68	0.24	0.33	0.22	0.24	0.39				
Ovary	0.41	0.31	0.40	0.13	0.56	0.52	0.22	0.39	0.27	0.07	0.39	0.38			
Skeletal Muscle	0.18	0.17	0.17	0.27	0.20	0.21	0.11	0.07	0.12	0.08	0.10	0.30	0.29		
Stomach	0.37	0.26	0.31	0.10	0.42	0.37	0.20	0.30	0.23	0.04	0.31	0.30	0.05	0.19	
Testis	0.76	0.60	0.68	0.37	0.92	0.89	0.46	0.68	0.55	0.23	0.65	0.73	0.24	0.51	0.22
