# Supplementary material for: Discovery of tissue-specific exons using comprehensive human exon microarrays
Source: Genome Biol. 2007 Apr 24;8(4):R64. doi: 10.1186/gb-2007-8-4-r64 (PMC1896007; doi:10.1186/gb-2007-8-4-r64)
Supplement: Additional data file 5 — (a) A histogram of the Pearson correlation coefficient of signal intensity across the 16 tissues for probesets belonging to the same exon cluster and probesets randomly selected from different exon clusters. (b) A table showing the median and average correlation and the total number and percent of exon clusters with correlations less than different values. [file gb-2007-8-4-r64-S5.pdf]

Additional File 5
Correlation of Exon Cluster Probeset Intensities

A

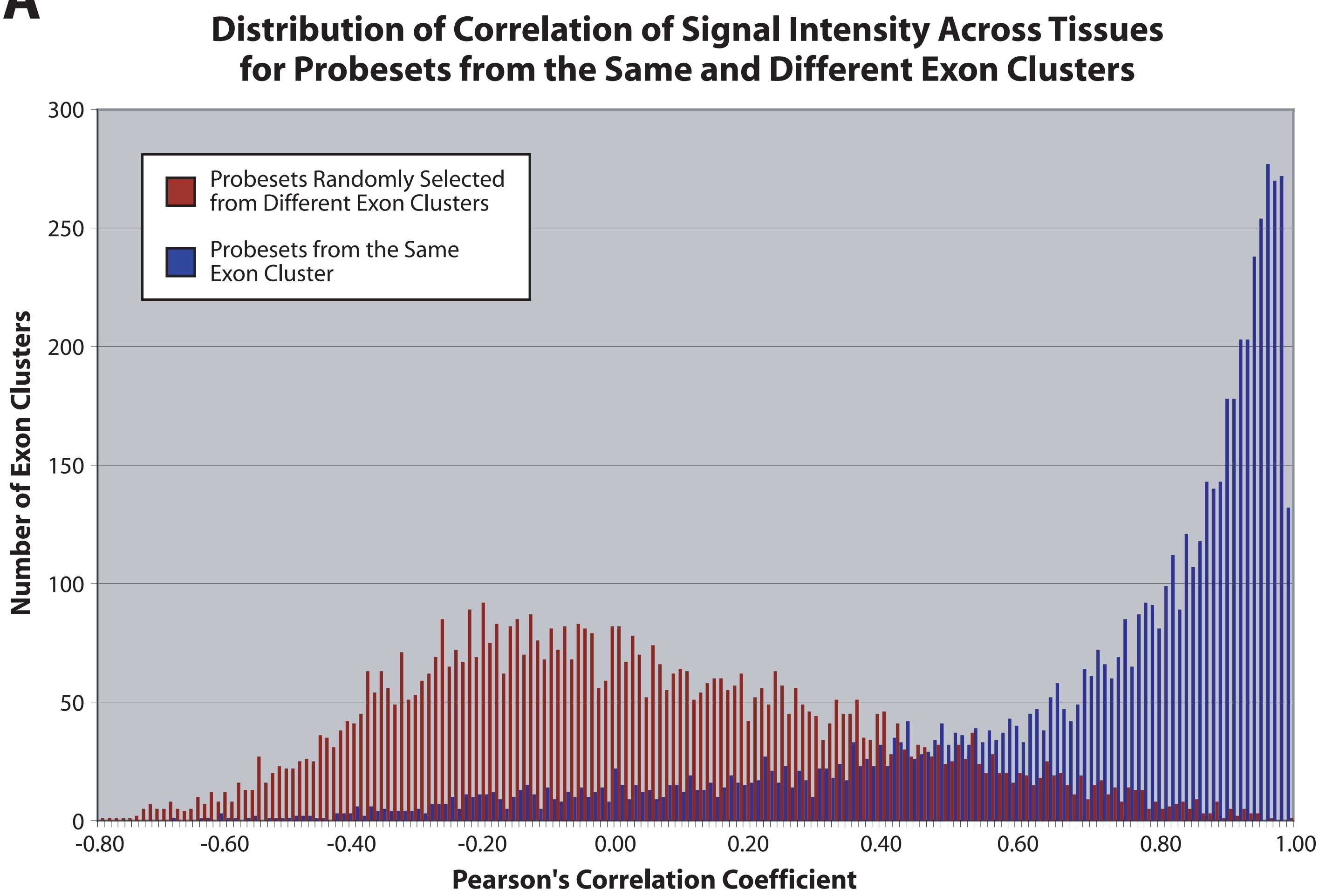

B

	Same Exon Cluster		Random Exon Cluster	
**MEDIAN CORRELATION**	**0.83**		**-0.01**	
**AVERAGE CORRELATION**	**0.701**		**0.030**	
**CORR <0**	341	5.39	3162	50.01
**CORR <.1**	469	7.41	3850	60.89
**CORR <.2**	616	9.74	4434	70.12
**CORR <.3**	803	12.69	4949	78.27
**CORR <.4**	1021	16.14	5374	84.99
**CORR <.5**	1344	21.25	5692	90.02
**CORR <.6**	1705	26.95	5940	93.94
**CORR <.7**	2156	34.08	6121	96.81
**CORR <.8**	2877	45.48	6240	98.69
**CORR <.9**	3978	62.88	6302	99.67
	**Count**	**%**	**Count**	**%**
