# Supplementary material for: Discovery of tissue-specific exons using comprehensive human exon microarrays
Source: Genome Biol. 2007 Apr 24;8(4):R64. doi: 10.1186/gb-2007-8-4-r64 (PMC1896007; doi:10.1186/gb-2007-8-4-r64)

Additional File 6
Robustness Analysis of Gene-Level Estimation

A

$$\text{Median Percent Deviation} = \text{Median}\left[\frac{\text{(altered gene-level estimate - original gene-level estimate)}}{\text{orginal gene-level estimate}} \times 100\right]$$

B

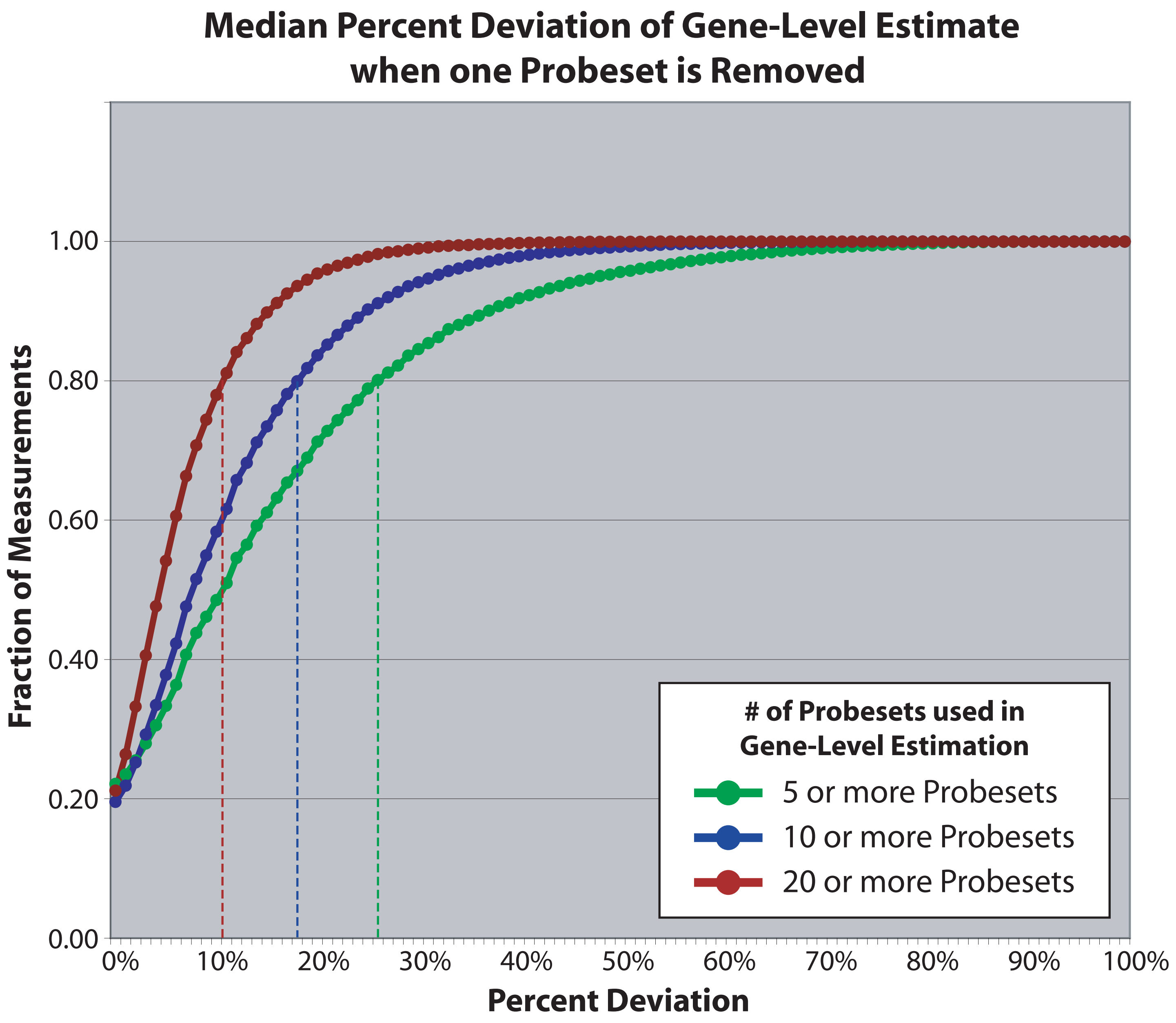

Supplement: Additional data file 6 — The robustness of the gene-level estimation method to alternative splicing was analyzed by simulating exon skipping events. The intensity of each probeset that was used in the gene-level estimate was systematically substituted for the background level and the gene-level estimate was re-computed. The deviation of the altered gene-level estimate from the original gene-level estimate was determined for every transcript cluster in each tissue sample. (a) The median percent deviation was calculated by taking the median of the difference of the altered and the original gene-level estimates divided by the original gene-level estimate all multiplied by 100. (b) A graph illustrating the median percent deviation of gene-level estimates for transcript clusters with 5 or more, 10 or more, and 20 or more probesets used in the calculation of the gene-level estimate. [file gb-2007-8-4-r64-S6.pdf]
