# Supplementary material for: Discovery of tissue-specific exons using comprehensive human exon microarrays
Source: Genome Biol. 2007 Apr 24;8(4):R64. doi: 10.1186/gb-2007-8-4-r64 (PMC1896007; doi:10.1186/gb-2007-8-4-r64)
Supplement: Additional data file 7 — Chip design information. [file gb-2007-8-4-r64-S7.pdf]

Additional Table 1 - Chip Design Information

Number of Chips	4 (A,B,C,D)
Feature Size	8um
Genome Version	NCBI Build 32, Nov 2002 (hg14)
Transcript Clusters	266,454
RefSeq / Ensembl Supported Transcript Clusters	19,221
Exon Clusters	1,032,926
Probe Selection Regions (PSRs)	1,440,489
Probesets	1,495,766
Probe Pairs	4,895,858
Unique Probes	9,665,769
Exon Cluster Median Length	149 nt
PSR Median Length	119 nt
PSR Minimum Length	17 nt
PSR Genome Coverage	286 MB
**Content Source**	**Probesets**
EnsGene	342,843
Genscan (+SubOpt)	326,514
SLAM	176,759
TwinScan	191,589
cDNA / EST	502,019
