# Supplementary material for: Discovery of tissue-specific exons using comprehensive human exon microarrays
Source: Genome Biol. 2007 Apr 24;8(4):R64. doi: 10.1186/gb-2007-8-4-r64 (PMC1896007; doi:10.1186/gb-2007-8-4-r64)
Supplement: Additional data file 8 — RNA sample information. [file gb-2007-8-4-r64-S8.pdf]

Additional Table 2 - RNA Sample Information

Tissue	Sample Name	ID	Lot #	Age	Gender	Group
Cerebellum	Br_Cerebellum_1	01A	A607140	26	Male	Brain
Cerebellum	Br_Cerebellum_2	01B	A607139	22	Male	Brain
Cerebellum	Br_Cerebellum_3	01C	A607138	28	Male	Brain
Corpus Callosum	Br_CorpusCallosum_1	02A	A608027	27	Male	Brain
Corpus Callosum	Br_CorpusCallosum_2	02B	A608028	26	Male	Brain
Corpus Callosum	Br_CorpusCallosum_3	02C	A608026	26	Male	Brain
Frontal Lobe	Br_FrontalLobe_1	03A	A607143	28	Male	Brain
Frontal Lobe	Br_FrontalLobe_2	03B	A607141	28	Male	Brain
Frontal Lobe	Br_FrontalLobe_3	03C	A607142	29	Male	Brain
Occipital Lobe	Br_OccipitalLobe_1	04A	A607146	22	Male	Brain
Occipital Lobe	Br_OccipitalLobe_2	04B	A607145	27	Male	Brain
Occipital Lobe	Br_OccipitalLobe_3	04C	A607144	28	Male	Brain
Parietal Lobe	Br_ParietalLobe_1	05A	A607147	27	Male	Brain
Parietal Lobe	Br_ParietalLobe_2	05B	A607148	21	Male	Brain
Parietal Lobe	Br_ParietalLobe_3	05C	A607149	24	Male	Brain
Temporal Lobe	Br_TemporalLobe_1	06A	A607150	26	Male	Brain
Temporal Lobe	Br_TemporalLobe_2	06B	A607151	27	Male	Brain
Temporal Lobe	Br_TemporalLobe_3	06C	A607152	33	Male	Brain
Spinal Cord	SpinaCord_1	07A	A607196	44	Male	NA
Spinal Cord	SpinaCord_2	07B	A607197	24	Male	NA
Spinal Cord	SpinaCord_3	07C	A607198	25	Male	NA
Adipose	Adipose_1	08A	A607134	24	Male	Non-Brain
Adipose	Adipose_2	08B	A607133	34	Male	Non-Brain
Adipose	Adipose_3	08C	A607132	36	Male	Non-Brain
Appendix	Appendix_1	09A	A607137	27	Male	Non-Brain
Appendix	Appendix_2	09B	A607136	21	Male	Non-Brain
Appendix	Appendix_3	09C	A607135	44	Male	Non-Brain
Heart	Heart_1	10A	A607163	29	Male	Non-Brain
Heart	Heart_2	10B	A607164	44	Male	Non-Brain
Heart	Heart_3	10C	A607165	27	Male	Non-Brain
Kidney	Kidney_1	11A	A607168	26	Male	Non-Brain
Kidney	Kidney_2	11B	A607167	26	Male	Non-Brain
Kidney	Kidney_3	11C	A607166	46	Male	Non-Brain
Liver	Liver_1	12A	A607169	23	Male	Non-Brain
Liver	Liver_2	12B	A607170	26	Male	Non-Brain
Liver	Liver_3	12C	A607171	30	Male	Non-Brain
Ovary	Ovary_1	13A	A606270	39	Female	Non-Brain
Ovary	Ovary_2	13B	A606272	45	Female	Non-Brain
Ovary	Ovary_3	13C	A606282	41	Female	Non-Brain
Skeletal Muscle	SkelMuscle_1	14A	A607190	44	Male	Non-Brain
Skeletal Muscle	SkelMuscle_2	14B	A607191	26	Male	Non-Brain
Skeletal Muscle	SkelMuscle_3	14C	A607192	24	Male	Non-Brain
Stomach	Stomach_1	15A	A607202	44	Male	Non-Brain
Stomach	Stomach_2	15B	A607203	24	Male	Non-Brain
Stomach	Stomach_3	15C	A607204	47	Male	Non-Brain
Testis	Testis_1	16A	A607057	29	Male	Non-Brain
Testis	Testis_2	16B	A607058	34	Male	Non-Brain
Testis	Testis_3	16C	A607060	27	Male	Non-Brain
