# Supplementary material for: Discovery of tissue-specific exons using comprehensive human exon microarrays
Source: Genome Biol. 2007 Apr 24;8(4):R64. doi: 10.1186/gb-2007-8-4-r64 (PMC1896007; doi:10.1186/gb-2007-8-4-r64)
Supplement: Additional data file 9 — RT-PCR primer sequences. [file gb-2007-8-4-r64-S9.pdf]

## Additional Table 3 - RT-PCR Primer Sequences

| Gene | Exons | Probeset ID(s) | Primer F | Primer R | Novel Exon(s) | GenBank Accession Number(s) | Figure | Comments |
|---|---|---|---|---|---|---|---|---|
| *PHLDB1* | Ex23/25 | 104550938 | AGGCCATTGAGGAAGTGTAC | CATCCATCCAGATACGCATG | | | Fig. 4A | Brain-enriched Exon |
| *SLC9A7* | Ex12/15 | 100381201, 100381206 | CTTCACTGTCTGGATCATTG | GTTTCCTCTGGCAGAATCTG | | | Fig. 4B | Brain-depleted Exon |
| *CLTA* | Ex4/7 | 104676545, 96693464, 96693460 | TAGAAGAATGGTATGCAAGAC | GGACGACTCGTCAATGTCA | | | Fig. 5 | Double Cassette |
| *RAB6IP2* | Ex18/20 | 95710993 | ACTACGAGGATGACCACTTC | TTGTCAGGTGTCCAATGTAC | | | Fig. 5 | |
| *TPD52* | Ex4/8 | 96804991 | ATCCCAGGCTGGACAGAAG | ATCACCACCAGCAGGCTTG | | | Fig. 5 | Double Cassette |
| *MYH10* | Ex15/16 | 94245141 | TTGCACCAGTCATCAGACAG | TTGCCATCAGCTTGGTGAG | 1 | DQ925667 | Fig. 5 | 1 Novel Brain-Enriched Exon |
| *ATP11B* | Ex28/31 | 98865728, 98865738, 98865742, 98865747 | CCTCATGGTTGTTACATGTC | GACTACATCTTCCTATAACTC | 1 | DQ925668 | Fig. 5 | 1 Novel Brain Enriched Exon; Ex29 is also a cassette |
| *EPB41L3* | Ex16/18 | 94015918 | AGACACTGCCGTAACGAATG | ACCACCTTCTCAGTGCTAAG | | | Fig. 5 | |
| *MINK1* | Ex17/19 | 83066970 | AATAAAGCCAAGCCCGACGA | CCAGAGTCCGCTCTTTCAG | | | Fig. 5 | |
| *WNK1* | Ex26/27 | 95712169 | CAGTGACAACCTCTATTCAG | GTGCAAGTCATCTGTGAATG | 2 | DQ925669 | Fig. 6 | 2 Novel Brain-Enriched Exons (one of which also has an alt 5'ss) |
| *WNK1* | Ex26/26b | 95712169 | CAGTGACAACCTCTATTCAG | CTGGTTGCAAGGACAGACT | | DQ925670, DQ925671, DQ925672 | Fig. 6 | |
| *OGDH* | Ex3/5 | 97454232 | CAGTGCAGTCACTCATCAG | TTGTCGAGGTCAGACTCATC | 1 | DQ925673, DQ925674 | Supp. Fig. 1 | 1 Novel Exon in addition to mutually exclusive Ex4a/4b |
| *LPHN2* | Ex15/18 | 100161769 | ACTATAAGAGCTATGGAACAG | GTGAAGAGATATGCCATCAC | 1 | DQ925675 | Supp. Fig. 1 | 1 Novel Brain-Enriched Exon |
| *DYM* | Ex12/14 | 93975279 | CCATTCAATACAACATGACTAG | AACTCAGCGAACCTCTCAAG | 1 | DQ925676 | Supp. Fig. 1 | 1 Novel Brain-Enriched Exon |
| *RAPGEF1* | Ex11/13 | 96442338 | ATGGAGGTATACGGCTTCAG | AGCCGACTCCAGAGCATCT | 4 | DQ925677, DQ925678, DQ925679, DQ925680, DQ925681, DQ925682, DQ925683, DQ925684 | Supp. Fig. 1 | 3 Novel Brain-Enriched Exons (one of which also has an alt 3'ss); Novel Exon 4 found in Ovary |
| *PLEKHA6* | Ex9/11 | 105717857 | CCGTGCCCGCATTTACTC | CACCTTGTTCTGCTCACACA | 3 | DQ925685, DQ925686, DQ925687, DQ925688, DQ925689, DQ925690, DQ925691 | Supp. Fig. 1 | 3 Novel Exons |
| *ANK2* | Ex47/49 | 98509599 | GCCACAGGAACCTGTCAAC | TTTCCATGCTGGTCCTTCAC | 1 | DQ925692, DQ925693 | Supp. Fig. 1 | 1 Novel Muscle-Enriched Exon |
| *FAT* | Ex26/27 | 98278505 | CTTTCCAAGAAGCCTCTAGAG | GTAGCCTCCAGGGTAATAGT | 1 | EF139845 | Supp. Fig. 4 | 1 Novel Brain-Enriched Exon |
| *CAMK2D* | Ex12/15 | 98337652, 98337647 | ACAACTATGCTGGCTACAAG | TTCACATCTTCATCCTCAATTG | | EF139846 | Supp. Fig. 4 | Novel arrangement of annotated exons |
| *PTPRK* | Ex15/16 | 97560338 | CACTCTGCATGCAGAAGATC | GGTAGCGAGGTACGTCTAG | | EF139847 | Supp. Fig. 4 | Novel arrangement of annotated exons |
| *RECK* | Ex2/5 | 96694017, 96694012 | TAACCAAATGTGCCGTGATG | CCAGTTCACAGCAGCCTAAG | | EF139848 | Supp. Fig. 4 | Novel arrangement of annotated exons |
| *UNC13B* | Ex10b/11 | 104676985 | ACCTCAACAAGTGCATCAAC | CCTTCCGGGAGCCACTGAG | | EF139849 | Supp. Fig. 4 | Novel arrangement of annotated exons |
| *ANK3* | Ex36/37 | 96180441, 96180431 | CAGATAGACGACAGAGCTTC | GGCCCAGGCACTGTAATCG | | EF139850 | Supp. Fig. 4 | Novel 5' ss |
| *ANK3* | Ex36/38 | 96180441, 96180431 | CAGATAGACGACAGAGCTTC | CTATTGCCATCCTGATATCTG | | EF139851 | Supp. Fig. 4 | Novel 5' ss |
| *SORBS2* | Ex15/16 | 98280250 | TGGACCTCCCAGGATCAAGC | CAGTCGTCGTTTAGGAGATC | | EF139852 | Supp. Fig. 4 | Novel 3' ss |
| *SLK* | Ex12/14 | 96295717 | AGAGCACACAAATCGCTTGC | GCACTCTCTCTCAATATTAGC | 1 | EF139853 | Supp. Fig. 4 | 1 Novel Exon Enriched in Non-Brain Tissues |
