# Supplementary material for: Discovery of tissue-specific exons using comprehensive human exon microarrays
Source: Genome Biol. 2007 Apr 24;8(4):R64. doi: 10.1186/gb-2007-8-4-r64 (PMC1896007; doi:10.1186/gb-2007-8-4-r64)
Supplement: Additional data file 10 — Additional RT-PCR primers. [file gb-2007-8-4-r64-S10.pdf]

## Additional Table 4 - Additional RT-PCR Primer Sequences

| Gene | Exons | Probeset ID(s) | Primer F | Primer R | Splicing Index P-Value Rank | SI Magnitude Rank | VALIDATEDED? |
|---|---|---|---|---|---|---|---|
| *GLCCI1* | Ex1/3 | 97494957 | GAGCAGCTCACCTGAGAGA | TCCTTACTGTGACGACTAC | 2 | 596 | |
| *TBC1D9B* | Ex17/20 | 97891655 | CAAGGTGCTCTACAAGCTAC | TTGCTCTTCCTGTGGTAGTG | 6 | 303 | |
| *ANK3* | Ex36/37 | 96180441, 96180431 | CAGATAGACGACAGAGCTTC | GGCCCAGGCACTGTAATCG | 7 | 400 | |
| *NPTN* | Ex1/3 | 94749260 | TTCTGCTAGGGAGGATGTC | TCTTTGTCCAGTAGCTGTATG | 8 | 628 | |
| *FEZ2* | Ex5/8 | 99307244 | TGGGAAGAATGAGAGAAGTCA | TTTCACTGTCCTCCTTCATG | 10 | 48 | |
| *TPD52L2* | Ex5/8 | 93583452 | GCAGGACAGAAGACTTCAG | TCGGTCCTCAAACGACTTG | 16 | 49 | |
| *ACTN4* | Ex6/8b | 93861390 | ACAGACCAGAGCTGATTGAG | TTCTCATCTGGCCTCAGAG | 21 | 233 | |
| *ZYFVE21* | Ex5/7 | 95078447 | ACATTTCCACCGTGCAGATC | CCACCACTGTCAGCTTCAG | 22 | 323 | |
| *D87470* | Ex2/5 | 95787339 | CCTAAGAACATGGCCTACAC | GCCACAGGAAGACGAGTTC | 24 | 312 | |
| *MICAL2* | Ex17/24 | 96051659, 96052696 | GCTGCTGGCCAAGTTTGAG | TCAGCCGTTCCATCACGTAC | 25 | 144 | |
| *MYO6* | Ex32/34 | 97796794 | TGCTCCAAAGTCTGTTACTG | TACTCTGAGGGTCTTTGTAC | 28 | 313 | |
| *TJAP1* | Ex7/8 | 97821142, 97821137 | AGGAGTTGGAGGACAAGCT | GCTCTTCCAGCTTGTTGATG | 29 | 292 | |
| *KIF1B* | Ex24/26 | 100266293 | AAAGCGAAACCACTGTGACT | CACCTTTCTCACTGACGATG | 31 | 187 | |
| *LAS1L* | Ex11b/12 | 105353418, 100363607 | TGCTGGTCTCGGAGAAGTC | | 33 | 364 | |
| *GDI1* | Ex2/4 | 105358575 | TCTGTGAACGGGAAGAAGGT | CACCTTGAAGTCCAGGTAG | 34 | 1100 | |
| *CLTB* | Ex4/6 | 105557570 | GCCAGAGTGAACAAGTAGAG | ACACATCTTTGCACTGCTTG | 36 | 72 | |
| *DUSP16* | Ex6/7 | 95521258 | TCGCTATCGCCTACATCATG | TTTGCTCTTTGGCCCTGATG | 45 | 484 | |
| *ARAF* | Ex11/12 | 100506032 | CGACACACGCTTCGACATG | ACTTGAGATCTCGGTGGATG | 47 | 432 | |
| *RECK1* | Ex2/5 | 96694017, 96694012 | TAACCAAATGTGCCGTGATG | CCAGTTCACAGCAGCCTAAG | 58 | 791 | |
| *THYN1* | Ex1/2 | 95715606 | GCAGTCGAGTCTGCAGAGT | AATGCCTCACCTGAGTTCTC | 60 | 799 | |
| *MAPK9* | Ex6a/7 | 104808736 | TGGTCCTCCATAAAGTCCTG | TTCCTCACAGTTGGCTGAAG | 62 | 268 | |
| *CADPS2* | Ex22/24 | 97162001 | TCCGCTATGTGGATCTCATG | GTTTAAGTCTTTGCTCTAAGTG | 63 | 130 | |
| *Y652* | Ex11/14 | 96016281 | GTCTGTAGAAGACTCTCAAG | GTCAGCCTGGGTACCATG | 68 | 33 | |
| *PRKCBP1* | Ex15/16 | 103802407 | GCTTTTACCGAAGGAGACTG | GGACACAAGGGTGCTCATG | 69 | 661 | |
| *CAMK2D* | Ex12/15 | 98337652, 98337647 | ACAACTATGCTGGCTACAAG | TTCACATCTTCATCCTCAATTG | 70 | 203 | |
| *SDCCAG8* | Ex13/16 | 105282261 | CGAATCTGAGCACCAACTG | TGCTTCATCGTCTCATGTAC | 75 | 722 | |
| *SORBS2* | Ex15/16 | 98280250 | TGGACCTCCCAGGATCAAGC | CAGTCGTCGTTTAGGAGATC | 340 | 8 | |
| *ACTN4* | Ex18/20 | 93860469 | CATCAGCCAGGAGCAGATG | CTCATGATGCGGTTGAACTC | 151 | 13 | |
| *UNC13B* | Ex10b/11 | 104676985 | ACCTCAACAAGTGCATCAAC | CCTTCCGGGAGCCACTGAG | 679 | 14 | |
| *EHBP1* | Ex7/9 | 99566362 | TATGAAGCAGGCTGACATTG | CAGCATCAGGATCATCAAATG | 107 | 17 | |
| *TPD52L2* | Ex5/8 | 103811692 | GCAGGACAGAAGACTTCAG | TCGGTCCTCAAACGACTTG | 126 | 32 | |
| *MLLT4* | Ex8/10 | 97705870 | AAACACTTGGAAGGCAAGAC | TGGCTTATCTCTAGAGTCAG | 279 | 34 | |
| *PTPRM* | Ex12/15 | 94095691 | TTTCCAAGCTGCTAGTAGAG | CTGTCGGGTGCTGCTCATG | 78 | 35 | |
| *NIBP* | Ex17/19 | 96746277 | CGACTACAGCCACGTGAAG | TCGGTGGAGTTGAAGACATC | 283 | 43 | |
| *FAT* | Ex26/27 | 98278505 | CCTGTCTGAAGTGCAGTCT | GGCATCCAATCTGATGTATC | 517 | 44 | |
| *SMTN* | Ex11/12 | 93390005 | GGGCAGTGTCACTCATGTC | TTCTGGTGCTTTGTTCACTC | 480 | 50 | |
| *ITSN2* | Ex16/18 | 99323041 | AACTGCATCTAAGCTGTCAG | CTTGCAGCCTCATCTTCTAG | 378 | 54 | |
| *BIN1* | Ex6/8 | 105077962 | AAGCTGGTGGACTACGACA | CTCCTGCAGATCCACATTC | 430 | 58 | |
| *APLP2* | Ex13/15 | 104546949 | GTGCAGATATGGACCAGTTC | TCTCTTCGGCACCGATCAG | 150 | 871 | |
| *RBM9* | Ex9/13 | 93318444 | CGGACAGTATATGGTGCAGT | CAGGCTGTGCATATCTGTAG | 206 | 783 | |
| *EPB41L3* | Ex15/17 | 94016008 | AGACACTGCCGTAACGAATG | ACCACCTTCTCAGTGCTAAG | 226 | 157 | |
| *PTPRK* | Ex15/16 | 97560338 | CACTCTGCATGCAGAAGATC | GGTAGCGAGGTACGTCTAG | 227 | 538 | |
| *GIT1* | Ex6/9 | 94213279 | GCCAATATGAGCTCACTGAC | TTGGCCAATTCGGATAAGTC | 229 | 141 | |
| *STAU* | Ex3/5 | 93520304 | CTTAACCTCTCAGAACTGAAC | GACTGCATCCGAGAGTAAG | 242 | 762 | |
| *KIAA0562* | Ex18/19 | 99950716 | TCTCATGCTGACAAGATGTG | CTCACTGCAACGGTAACAC | 249 | 396 | |
| *EPB41L1* | Ex12/14 | 93626410 | GTCAGCGAGAACCATGATG | CTTCAGCAAGACATCTTCTG | 337 | 1066 | |
| *SPIRE1* | Ex8/9 | 94005738 | CCAGAGGAGATTAGACGTAG | GCACCTGCTGTGAGGTACT | 345 | 118 | |
| *HM13* | Ex11/13 | 93637662 | GGCCAAGGGAGAAGTGACA | GATGCTGATGCCTCTGTTC | 611 | 428 | |
| *EPB41L3* | Ex11/14 | 94015893 | CCTTACTTTGAACGCTCATC | CCGTGCGCTCACTGTCAG | 703 | 548 | |
| *EPB41L3* | Ex14/16 | 94015918 | GACAGTGAGCGCACGGAC | TTACGGCAGTGTCTGTTGAG | 753 | 250 | |
| *MARK4* | Ex15/18 | 93843376 | AACCTCTTCACCAAGCTGAC | GTTTGATCCCAAGGTAGATG | 860 | 343 | |
| *APLP2* | Ex6/8 | 104546909 | TGAGGAGAATCCTACTGAAC | GAGCATGCTCATTATCATCTG | 929 | 1046 | |
| *DCNI2* | Ex5/7 | 105158206 | TGTGAGCACTCCAAGTGAAG | AGTTTCCTTTGTATACGTGAC | | | |
