# Supplementary material for: Discovery of tissue-specific exons using comprehensive human exon microarrays
Source: Genome Biol. 2007 Apr 24;8(4):R64. doi: 10.1186/gb-2007-8-4-r64 (PMC1896007; doi:10.1186/gb-2007-8-4-r64)
Supplement: Additional data file 11 — Normalization control genes. [file gb-2007-8-4-r64-S11.pdf]

Additional Table 5 - Normalization Control Genes

GENE	TRANSCRIPT_CLUSTER	DESCRIPTION
TARDBP	1_10782225-10795095	TAR DNA binding protein
ILF2	1_150851307-150860271_RC	interleukin enhancer binding factor 2
SLC39A1	1_151148370-151231385_RC	Zinc transporter ZIP1
ARF1	1_225244992-225261541	ADP-ribosylation factor 1
GUK1	1_225302622-225311287	Guanylate kinase
RPL11	1_23487769-23492390	60S ribosomal protein L11
KHDRBS1	1_31981955-32028923	KH domain containing, RNA binding, signal transduction associated 1
PSMB2	1_35496402-35534805_RC	Proteasome subunit beta type 2
ATP6V0B	1_43853905-43857260	Vacuolar ATP synthase 21 kDa proteolipid subunit
PARK7	1_7731339-7754885	DJ-1 protein (Oncogene DJ1)
ALS2CR17	2_204175240-204293644	neurobeachin-like 1
C2orf24	2_220239158-220245271_RC	hypothetical protein LOC27013
NEDD5	2_242575525-242614182	Septin 2 (NEDD5 protein homolog)
RPS7	2_3187378-3193019	40S ribosomal protein S7
RPS27A	2_55434358-55437279	Ubiquitin
STARD7	2_96335353-96359319_RC	StAR-related lipid transfer protein 7
U520_HUMAN	2_96424830-96456040_RC	U5 small nuclear ribonucleoprotein 200 kDa helicase
RPL34	4_110000543-110010388	60S ribosomal protein L34
RPL9	4_39352927-39357717_RC	ribosomal protein L9
HNRPD	4_83732862-83753544_RC	Heterogeneous nuclear ribonucleoprotein D0
HINT1	5_130571198-130577252_RC	Histidine triad nucleotide-binding protein 1 (Adenosine 5'- monophosphoramidase)
IK	5_140055884-140070565	Red protein (RER protein)
ATP6V0E	5_172391686-172442823	Vacuolar ATP synthase subunit H
CANX	5_179235655-179267584	Calnexin precursor
SNX3	6_108559405-108628033_RC	Sorting nexin 3 (SDP3 protein)
CBX3	7_25983371-25999238	Chromobox protein homolog 3 (Heterochromatin protein 1 homolog gamma)
FNTA	8_42928844-42958291	Protein farnesyltransferase/geranylgeranyltransferase type I alpha subunit
RPL30	8_99010528-99014356_RC	60S ribosomal protein L30
RPS6	9_19366254-19370235_RC	ribosomal protein S6
HNRPK	9_82040211-82052638_RC	Heterogeneous nuclear ribonucleoprotein K (hnRNP K)
SMNDC1	10_111717385-111729284_RC	Survival of motor neuron-related splicing factor 30
GDI2	10_5811192-5859379_RC	Rab GDP dissociation inhibitor beta
EIF4G2	11_10782910-10794780_RC	Eukaryotic translation initiation factor 4 gamma 2
ZNF259	11_116186928-116196400_RC	Zinc-finger protein ZPR1
C11orf58	11_16724503-16741882	small acidic protein
SYVN1	11_64643358-64677363_RC	synoviolin 1 isoform b
CFL1	11_65397642-65401164_RC	Cofilin, non-muscle isoform
SART1	11_65504560-65522967	squamous cell carcinoma antigen recognized by T cells 1
TAF10	11_6596382-6597754_RC	Transcription initiation factor TFIID subunit 10
EIF3S5	11_7972746-7982027	Eukaryotic translation initiation factor 3 subunit 5
SART3	12_107418824-107457404_RC	squamous cell carcinoma antigen recognized by T cells 3
SFRS9	12_119273523-119319511_RC	Splicing factor, arginine/serine-rich 9
ANAPC5	12_120116781-120202156_RC	Anaphase promoting complex subunit 5 (APC5)
ARF3	12_47616259-47637577_RC	ADP-ribosylation factor 3
SLC25A3	12_97489871-97498246	Phosphate carrier protein, mitochondrial precursor
DAD1	14_21023935-21048257_RC	Defender against cell death 1 (DAD-1)
ERH	14_67836886-67855062_RC	Enhancer of rudimentary homolog
YY1	14_98695143-98734845	Transcriptional repressor protein YY1 (Yin and yang 1)
RNPS1	16_2208773-2258038_RC	RNA-binding protein S1, serine-rich domain
TCEB2	16_2821336-2827218_RC	Transcription elongation factor B polypeptide 2
KARS	16_75441183-75461130_RC	Lysyl-tRNA synthetase
COX4I1	16_85614675-85622087	Cytochrome c oxidase subunit IV isoform 1, mitochondrial precursor
PRPF8	17_1760515-1794691_RC	U5 snRNP-specific protein
USP22	17_21064939-21109102_RC	Ubiquitin carboxyl-terminal hydrolase 22
RPL27	17_41523611-41528502	60S ribosomal protein L27
MYST2	17_48340702-48381096	PREDICTED: similar to MYST histone acetyltransferase 2; histone acetyltransferase
SPAG7	17_5029164-5071674_RC	sperm associated antigen 7
DDX5	17_63045835-63052508_RC	Probable RNA-dependent helicase p68
DULLARD	17_7347723-7355828_RC	dullard homolog
RPL17	18_45267716-45270838_RC	ribosomal protein L17
NARS	18_53416875-53438009_RC	Asparaginyl-tRNA synthetase, cytoplasmic
C19orf50	19_18529604-18541188	hypothetical protein LOC79036
OAZ1	19_2220520-2224487	Ornithine decarboxylase antizyme
EEF2	19_3927054-3936451_RC	Elongation factor 2 (EF-2)
CAPNS1	19_41322757-41333095	Calpain small subunit 1 (CSS1)
ZNF146	19_41411488-41421506	Zinc finger protein OZF
RPL28	19_60589112-60628852	60S ribosomal protein L28
HNRPM	19_8415651-8478786	Heterogeneous nuclear ribonucleoprotein M (hnRNP M)
C22orf28	22_31108123-31132787_RC	hypothetical protein LOC51493
EIF3S7	22_35149936-35168522_RC	Eukaryotic translation initiation factor 3 subunit 7
NONO	X_69370258-69387815	54 kDa nuclear RNA- and DNA-binding protein
